# Moral and perceptual conflict processing are implemented by distinct brain activation and functional connectivity patterns

**DOI:** 10.1371/journal.pbio.3004025

**Published:** 2026-10-05

**Authors:** Ruida Zhu, Marcus Grueschow, Marius Moisa, Chao Liu, Christian C. Ruff

**Affiliations:** 1 Department of Psychology, Sun Yat-sen University, Guangzhou, China; 2 Zurich Center for Neuroeconomics, Department of Economics, University of Zurich, Zurich, Switzerland; 3 Healthy Longevity Center, University of Zurich, Zurich, Switzerland; 4 State Key Laboratory of Cognitive Neuroscience and Learning & IDG/McGovern Institute for Brain Research, Beijing Normal University, Beijing, China; 5 Beijing Key Laboratory of Safe AI and Superalignment, Beijing, China; 6 Beijing Institute of AI Safety and Governance, Beijing, China; 7 University Research Priority Program ‘Adaptive Brain Circuits in Development and Learning’ (URPP AdaBD), University of Zurich, Zurich, Switzerland; Oxford University, UNITED KINGDOM OF GREAT BRITAIN AND NORTHERN IRELAND

## Abstract

While humans resemble other primates in terms of motor and perceptual abilities, our behavior is uniquely characterized by morality—the ability to choose our actions based on specific ethical values. The evolutionary origins and neurobiological basis of moral behavior are debated intensely, with opposing proposals about whether moral decisions rely on domain-general decision-making neural circuitry (neural common currency hypothesis) or on unique neural processes that were shaped by evolutionary pressures associated with life in groups (social brain hypothesis). Here we examine the potential overlap between moral and perceptual decision-making, by combining functional magnetic resonance imaging with computational modeling of behavior in a novel task requiring human participants to make alternating moral and perceptual choices on identical stimuli. At the algorithmic level, we found that moral and perceptual choices were well fit by the same sequential sampling model, which allowed us to quantify and directly compare conflicts between the choice alternatives for both types of decision-making. At the implementational level, moral and perceptual choice conflicts were represented by distinct neural activity patterns; in fact, neural signals in some brain areas (e.g., temporoparietal junction) encoded the strength of the two types of conflict in opposite directions. Our findings suggest that while similar computational decision mechanisms underlie both moral and perceptual choices, their neural implementation is fundamentally different, supporting the social brain hypothesis.

## Abstract 2

Moral decisions may share computational mechanisms with perceptual decisions, but whether they rely on common or distinct neural systems remains debated. This study shows that moral and perceptual choices follow similar evidence-accumulation computations, yet engage fundamentally different neural representations, particularly in social brain regions such as the temporoparietal junction.

## Introduction

Morality distinguishes humans from other animals [1,2]. In human society, moral choices are not only crucial for maintaining individual well-being [3,4] but are also essential for sustaining large-scale social cooperation, a milestone of human civilization [5–8]. Questions about the origins of morality have long captured the interests of philosophers and scientists, in particular the question whether moral decision-making constitutes a unique, encapsulated domain of human cognition or rather a natural extension of domain-general cognition that ties in seamlessly with other mental abilities [9–13]. Empirically, one way to address this question is to examine the extent to which moral and non-moral choices, such as basic perceptual decisions, are guided by shared neural mechanisms. Answering this question is important for understanding the uniqueness of moral decision-making and may also have broader implications for the treatment of mental disorders [14,15] and the development of artificial intelligence with moral capabilities [16].

Existing neuroscientific accounts offer competing predictions about whether moral choices, such as judging whether killing is morally worse than stealing, and perceptual choices, such as judging whether a motorbike is larger than a mountain bike, rely on domain-general or domain-specific neural mechanisms. On the one hand, the neural common currency hypothesis proposes that neural signals in several brain areas integrate all kinds of information on a common value scale for guiding choices [17–21]; this would enable individuals to compare and choose between seemingly incommensurable options (e.g., saving money for oneself versus protecting others from pain) [22]. In support of this view, studies have suggested that several value-related brain regions, such as the ventromedial prefrontal cortex (VMPFC), represent both moral and material value information [23,24]. By logical extension, it appears plausible that some brain sites may encode both moral and perceptual information on a common neural scale. This assumption dovetails with empirical findings of psychological studies on moral metaphors, showing that moral concepts are often naturally mapped onto spatial concepts (e.g., “moral is big”, “immoral is small”) [25,26].

On the other hand, the social brain hypothesis emphasizes that moral choices may rely on domain-specific neural mechanisms that have evolved to specifically process the social information that is ubiquitous during complex social interactions [27]. This view has gathered support from neuroanatomical studies showing that neocortex size correlates with the social aspects of the environment but not with the non-social aspects [28–31]. It is also supported by task-based neuroimaging studies indicating that social (other-oriented) and non-social (self-oriented) signals (e.g., prediction errors and rewards) can be implemented in different subregions within the prefrontal cortex [32–35]. As morality is often considered to be a collective solution to problems occurring in social interactions [36,37], moral choices related to concerns about others are naturally social. In the view of the social brain hypothesis, moral (i.e., social) choices may thus rely on domain-specific neural processes not shared with non-social (e.g., perceptual) choices.

While several lines of research have examined this issue via the neural correlates of moral information processing, they have not yet been able to provide conclusive evidence to support or contradict the neural specificity of moral choices. For example, in studies comparing brain activity between moral and non-moral reasoning using neuroimaging techniques (see a review [13]), it has been notoriously difficult to ensure that the only difference between the conditions is the information type (moral versus non-moral information) rather than other aspects of the tasks, such as visual stimuli, motor demands, and basic cognitive processes. This makes it hard to evaluate whether any neural differences resulted from condition differences in cognitive processes (e.g., the moral condition contained conflict information processing but the non-moral condition did not) or whether the same cognitive processes (e.g., conflict information processing) are engaged but have different neural representation across different conditions. Only the latter case may provide direct support for the neural specificity of moral choices [20]. This difficulty in creating comparable moral and non-moral conditions may have led to mixed findings reported in the literature: Some studies have revealed that moral choices compared to perceptual, factual, or grammatical choices induced stronger activation in the medial prefrontal cortex (MPFC), lateral prefrontal cortex (LPFC), posterior cingulate cortex (PCC), and temporoparietal junction (TPJ) [38–42]; whereas other studies have reported that activity in the very same brain regions (MPFC, LPFC, precuneus, and TPJ) was higher for factual or grammatical choices compared to moral choices [43–45].

In the second line of research on this issue, studies have examined moral choices by combining neuroimaging techniques with computational models (see a review [23]). This approach enables more precise characterization of the cognitive processes underlying moral choices and clarification of where in the brain they are implemented. For example, several studies have successfully applied computational models originally developed for value-based choices to moral research (e.g., [46,47]). These studies have shown that model-defined moral value computation processes correlate with both the classical valuation brain system (e.g., VMPFC, striatum), brain areas outside of the classical valuation brain system (e.g., LPFC, TPJ, and insula), and functional coupling among these two sets of areas [22,48–57]. However, most studies along this research line presented choices between obeying moral norms versus gaining monetary rewards. Thus, participants had to explicitly trade off moral norms against monetary rewards, which may have led them to consider moral information within an economic framework. Additionally, few studies in this line created a non-moral control condition that would have made it possible to directly compare moral and non-moral choices within the same participants. Although one may use the neural findings of other non-moral studies as a comparison, the unmatched tasks and individual differences may cover nuanced but crucial neural differences. Therefore, it is difficult to judge based on the existing research whether moral choices, especially those that do not involve explicit trade-offs between moral norms and monetary self-interest, recruit domain-specific neural processes as compared to non-moral choices.

A useful framework to advance the study of neural distinctions and commonalities between moral and non-moral choices is Marr’s three levels of analysis of information-processing systems [58]. In this framework, one can characterize an information-processing system at (1) the computational level to determine the goal of the system (e.g., solving moral or non-moral problems), (2) the algorithmic level to specify the rules (e.g., algorithms and models) the system uses, or (3) the implementational level to describe the physical (e.g., neural) realization of the system’s rules. Many of the limitations in the previous research described above may relate to a narrow focus on the implementation level, without a precise characterization at the computational and algorithmic levels. Here we argue that the following points are essential for improving the methodology of testing the neural specificity of moral choices: (1) The tasks of the moral and non-moral conditions should be clearly separable at the computational level: moral choices and non-moral choices need to be used for comparison; (2) the algorithmic level (i.e., the cognitive processes involved) should be matched between the two domains, to ensure that behavioral and neural effects reflect only differences in the type of information that needs to be processed, rather than in the underlying cognitive processes themselves; and (3) the implementational level (i.e., the neural basis of these cognitive processes) needs to be informed by the first two steps, ensuring a mechanistic link to the model of information processing. It may thus be optimal to adopt experimental designs in which moral and non-moral choices share the same cognitive processes (i.e., holding the algorithmic level constant), and then examine whether the shared cognitive processes are differently implemented in the brain during moral and non-moral choices (see, e.g., [20]).

Following this logic, we developed a novel paradigm that allows participants to make binary moral and perceptual choices based on the same set of pictures, using an identical form of motor outputs. Participants completed this paradigm during fMRI, allowing us to compare the neural processes involved in information processing during the two types of choices. Past research has demonstrated a systematic influence of conflict levels (i.e., the complement of the evidence) on the speed and accuracy of choices [59,60]. Moreover, this influence and the associated decision processes can be effectively decomposed by a prominent sequential sampling model with clear algorithmic description, the drift-diffusion model (DDM), in both moral [61–63] and perceptual [60,64] domains. Based on this knowledge, we designed our stimulus material to make decision conflict processing at the algorithmic level fully comparable between moral and perceptual choices, by manipulating the conflict levels during both moral and perceptual choices. Confirming the algorithmic similarity, our computational modeling results show that the same drift-diffusion model can capture the speed and accuracy of moral and perceptual choices, and that the experimentally manipulated conflict level is indeed a key cognitive component influencing both types of choices.

At the implementational level, we could therefore test the neural specificity of moral conflict processing by comparing the neural correlates of moral and perceptual conflict levels using functional magnetic resonance imaging (fMRI). Moral-specific neural activity refers to responses that are stronger for moral conflicts than for perceptual conflicts. Our activation and psychophysiological interaction analysis showed that the moral and perceptual conflict levels were represented by distinct brain regions and different patterns of functional connectivity. Intriguingly, some areas (e.g., TPJ) even showed opposite response profiles to moral and perceptual conflict. These findings support the neural specificity of moral conflict processing and are consistent with the social brain hypothesis.

## Results

### Behavioral paradigm

We recorded fMRI data from healthy participants (see Materials and methods for details) performing our novel choice task. Before the choice task, participants were asked to provide ratings for a set of social pictures outside of the fMRI scanner using an on-screen slider scale. For moral and perceptual ratings, participants indicated “how severe is the moral violation depicted by the picture” and “how much in percent does the picture cover the gray background within the white frame”, respectively (S1 Fig). Participants could accurately judge the picture sizes, as demonstrated by a strong correlation between perceptual ratings and actual area sizes of the pictures (i.e., the percentage of a picture’s pixels that was distinct from the background color) (fixed effect: *β* = 0.86 ± 0.009, *T*(53.01) = 97.88, *P* < 0.001, hierarchical linear mixed-effect regression; S2 Fig).

Based on the ratings provided by each participant, we generated trials of moral and perceptual choices with four different conflict levels (see Materials and methods for more details). Crucially, the participants’ moral ratings were unrelated to the perceptual ratings (fixed effect: *β* = 0.02 ± 0.016, *T*(3869) = 1.29, *P* = 0.197, hierarchical linear mixed-effect regression; S3 Fig) and the pictures’ actual area sizes (fixed effect: *β* = −0.01 ± 0.016, *T*(3869) = −0.68, *P* = 0.498, hierarchical linear mixed-effect regression; S4 Fig), confirming that we could examine moral and perceptual choices in an independent fashion. We note that we exerted considerable effort to construct a stimulus set that independently varied the moral and perceptual information (i.e., the moral wrongness and area size), including careful selection and editing of the pictures and conducting seven pilot experiments (see Materials and methods).

Afterwards, participants performed the choice task in which they alternated between making moral or perceptual choices on the same set of pictures, involving an identical form of motor outputs, during fMRI scanning. In each trial, participants saw a pair of pictures and chose one of them according to the instructions for the condition they were in (cued by a central letter) (Fig 1A). In the moral condition, participants indicated “which picture depicts a more severe moral violation” (i.e., moral choice), whereas in the perceptual condition, participants indicated “which picture covers more gray background within the white frame” (i.e., perceptual choice). Moral conflict value was defined as 100% minus the absolute difference in “severity of moral violation” ratings between the two pictures (i.e., 100% − |moral rating_1_ − moral rating_2_|), such that higher values indicate greater moral conflict. Perceptual conflict value was defined in a similar manner, based on 100% minus the absolute difference in area size between the two pictures (i.e., 100% − |area size_1_ − area size_2_|) (see Materials and methods for more details). A correct moral (or perceptual) choice was defined as a response in which participants chose the picture with a higher moral rating (or with a larger area size). Moral and perceptual blocks contained 7–9 trials and were presented in alternation (Fig 1B).

**Fig 1:**
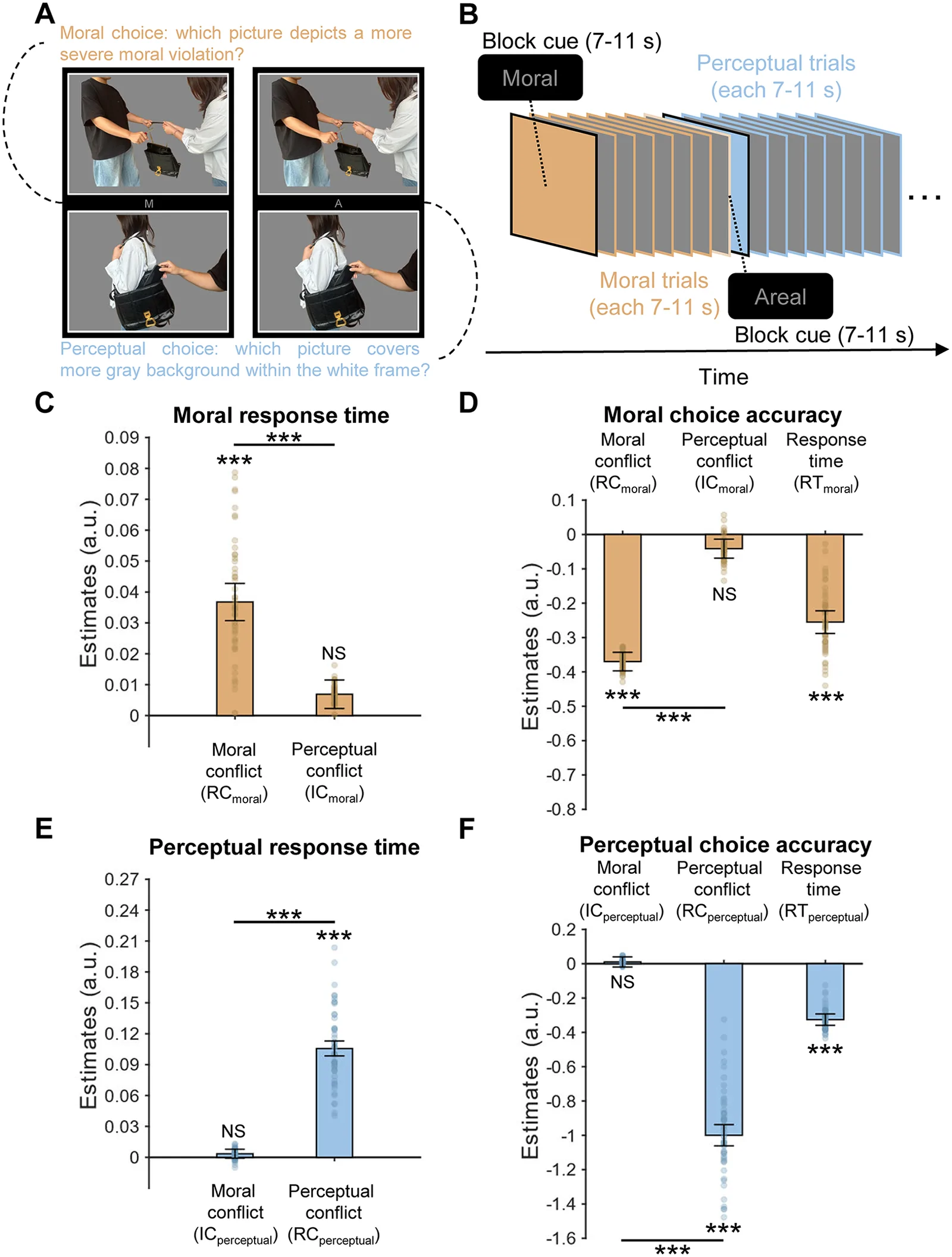
Behavioral paradigm and separation of moral and perceptual choices. **(A)** Example stimuli in the choice task. In a trial, participants were always cued about which type of condition they were in by a central letter (M = moral condition, A = perceptual condition). In the moral condition, participants chose which picture depicted a more severe moral violation. In the perceptual condition, they chose which picture covered more gray background. **(B)** Structure of blocks. Moral and perceptual blocks (containing 7–9 trials) were presented alternately. Each block started with a block cue (7 to 11 s) indicating the type of trials in it (Moral = moral condition, Areal = perceptual condition). Orange and blue squares with black frames represent moral and perceptual block cues, respectively. Gray squares with orange and blue frames represent trials (7 to 11 s) in the moral and perceptual conditions, respectively. **(C, E)** Response time (RT) was only affected by the task-relevant conflict (RC, i.e., the moral conflict for the moral choice (RC_moral_); the perceptual conflict for the perceptual choice (RC_perceptual_)) but not by the task-irrelevant conflict (IC, i.e., the perceptual conflict for the moral choice (IC_moral_); the moral conflict for the perceptual choice (IC_perceptual_)) in both conditions. **(D, F)** Choice accuracy was only affected by the task-relevant conflict but not by the task-irrelevant conflict in both conditions. The effects of the task-relevant conflict were independent of the influence of response time. RT_moral_, response time for the moral choice; RT_perceptual_, response time for the perceptual choice. **(C–F)** The task-relevant conflict had stronger effects on response time and choice accuracy than the task-irrelevant conflict in both conditions. Bar height represents the group-level effect from the mixed-effect regressions, while each dot indicates the estimate of an individual participant. Error bars represent the standard errors of the estimates from the mixed-effect regressions. ***P < 0.001. NS, not significant. The underlying data for this figure are available in the “Data” folder of our OSF repository.

### Separation of moral and perceptual choice processes

We first established that our paradigm allowed clear identification and separation of the computational processes underlying moral and perceptual choices. For this, we ran four regression analyses. In two of them, we regressed participants’ response time (RT) in the moral and perceptual conditions onto task-relevant conflict (RC) and task-irrelevant conflict (IC) levels (fixed-effect regressors: RC and IC); in the other two, we regressed participants’ choice accuracies in the moral and perceptual conditions onto task-RC, task-irrelevant conflict, and response time (fixed-effect regressors: RC, IC, and RT). In all analyses, we included participant-specific random constants and random slopes for each fixed effect (see Materials and methods for more details). Task-relevant conflict referred to the moral conflict for the moral choice (RC_moral_) and perceptual conflict for the perceptual choice (RC_perceptual_), while task-irrelevant conflict referred to the perceptual conflict for the moral choice (IC_moral_) and moral conflict for the perceptual choice (IC_perceptual_) (see S5 Fig for a more detailed explanation). These analyses confirmed that only the task-relevant conflict affected the speed and accuracy of participants’ choices, while task-irrelevant conflict was not associated with the speed and accuracy (Fig 1C, 1D, 1E, and 1F; S1, S2, S3, and S4 Tables). Direct comparisons showed that task-relevant (vs. task-irrelevant) conflict had stronger effects on choice accuracy and response time, in both the moral (RT: *χ*²(1) = 16.03, *P* < 0.001; choice accuracy: *χ*²(1) = 70.78, *P* < 0.001) and the perceptual (RT: *χ*²(1) = 128.84, *P* < 0.001; choice accuracy: *χ*²(1) = 228.44, *P* < 0.001) conditions. Thus, participants followed our instructions and used moral and perceptual conflict information for making the corresponding choices in an independent fashion.

### Moral and perceptual choices are well captured by the same computational decision model

To examine moral and perceptual choices at the algorithmic level, we tested four variants of drift-diffusion models linking choice accuracy and response time to the task-relevant conflict between the choice options (defined as the complement of the evidence; task-relevant conflict = 100% − evidence; see Materials and methods). The models differed as to whether the drift rate was affected by the task-relevant conflict and whether the threshold collapsed over time (see Materials and methods). We adopted accuracy coding such that boundaries correspond to a correct and incorrect choice and that a more positive drift rate represents stronger integration of evidence and thus higher efficiency in solving conflict. In the model comparison, the winning model for both the moral and perceptual conditions (Model 2, Fig 2A and 2D) has a constant threshold and a drift rate that depends on the task-relevant conflict. Posterior predictive checks show that the winning model provides a good fit to both types of choices (Fig 2B, 2C, 2E, and 2F). The findings suggest that while the task-relevant conflict, which emerges from different types of information for the two choice types, is a key cognitive component for both types of choices in our task, it is processed by a similar decision mechanism for both moral and perceptual choices.

**Fig 2:**
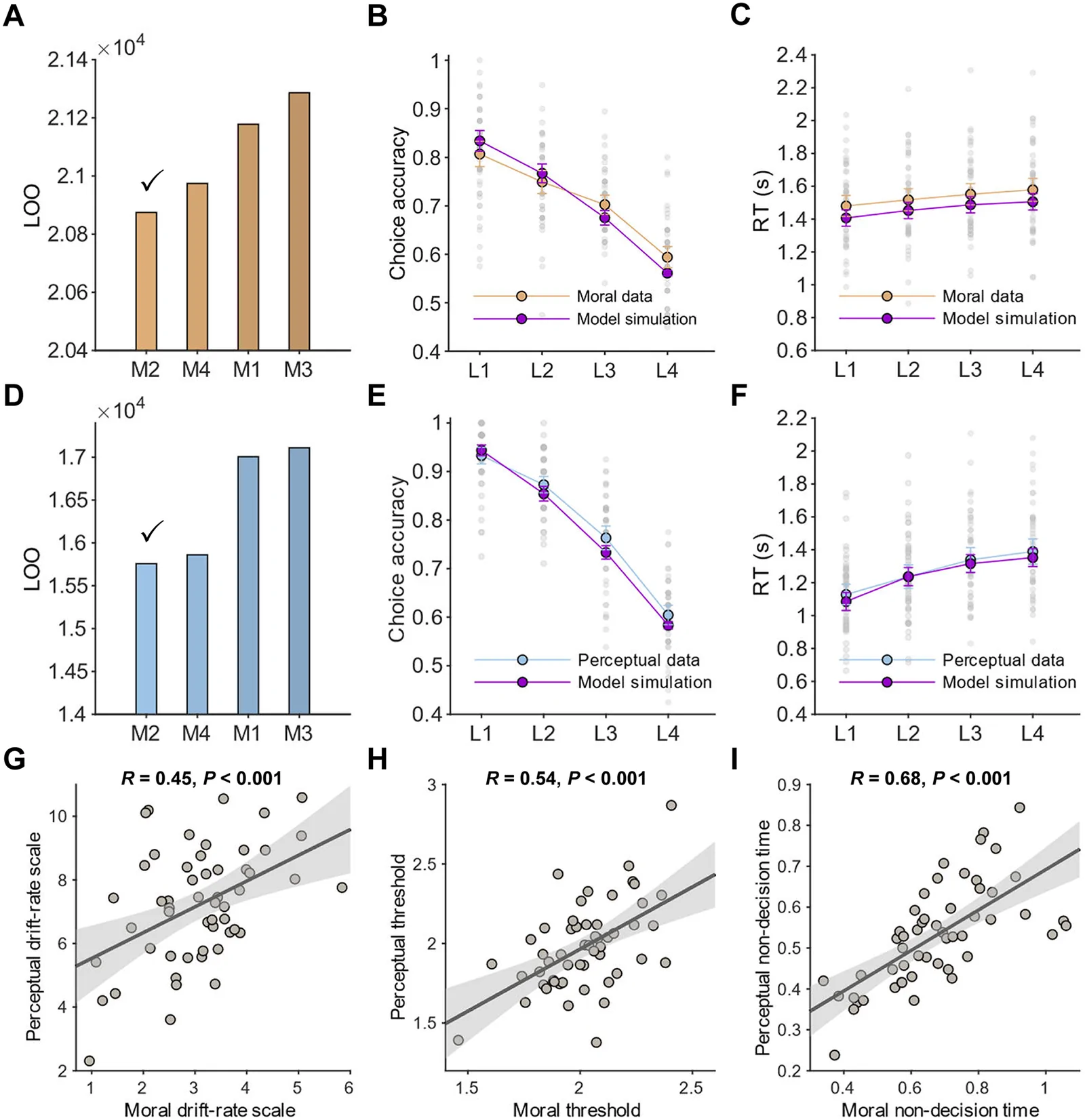
Moral and perceptual choices are captured by the same drift-diffusion model in which task-relevant conflict modulates drift rate. **(A, D)** Model comparison between four drift-diffusion models in the moral (**A**) and perceptual (**D**) conditions. Model 2, which assumes the drift rate is modulated by task-relevant conflict and the threshold is constant over time, won in both conditions based on the leave-one-out cross-validation score (LOO). M1 to M4 represent Models 1 to 4. ✓ denotes the winning model. (**B, C, E, F**) Posterior predictive checks: Model simulations of the winning model reproduced the behavioral patterns of choice accuracy and response time (RT) of the moral (**B, C**) and perceptual (**E, F**) choices. The mean values and 95% confidence intervals (error bars) of the model simulations, moral data, and perceptual data are presented in purple, orange, and blue. The values for individual participants’ choice accuracy and response time are shown in gray. L1 to L4, conflict level 1 to conflict level 4. (**G–I**) Correlations between the drift-rate scale **(G)**, threshold **(H)**, and non-decision time (**I**) of the moral and perceptual choices are displayed. Each gray dot depicts a participant’s value. Each black line represents the least squares fit with shading showing the 95% confidence interval. The underlying data for this figure are available in the “Data” folder of our OSF repository.

Dovetailing with previous findings [60,63], our modeling results therefore suggest algorithmic similarity between moral and perceptual choices. To further characterize this similarity, we tested whether the parameters capturing the idiosyncrasies of the participants’ choice processes in the moral and perceptual conditions correlate with each other. Our results show that the winning model’s parameter estimates for the moral and perceptual choices were significantly correlated with each other (drift-rate scale: *R* = 0.45, *P* < 0.001, Pearson correlation; threshold: *R* = 0.54, *P* < 0.001, Pearson correlation; non-decision time: *R* = 0.68, *P* < 0.001, Pearson correlation; Fig 2G, 2H, and 2I). This reveals commonalities in terms of information processing speed (indicated by drift-rate scale), choice caution (indicated by threshold), and sensory delay/movement initiation (indicated by non-decision time) between the moral and perceptual choices, thus further supporting that moral and perceptual choices could be described by a common algorithmic framework.

### Distinct neural representation of task-relevant conflict during moral and perceptual choices

After confirming the algorithmic similarity between moral and perceptual choices, we focused on how these choice mechanisms are implemented in the brain. Our primary analyses examined how the key information processed by the decision mechanism, namely task-relevant conflict, was neurally represented during moral and perceptual choices. Specifically, univariate activation analyses examined whether moral and perceptual task-relevant conflicts were associated with distinct regional activation patterns, whereas psychophysiological interaction (PPI) analyses examined whether conflict-dependent functional connectivity differed across moral and perceptual domains. If moral and perceptual conflict recruited overlapping activation or connectivity patterns, this would support a domain-general neural implementation of conflict processing; by contrast, distinct activation or connectivity patterns would support domain-specific neural implementation. Additional analyses of response-time-related activity, task-irrelevant conflict, chosen value, nonlinear conflict effects, and associations between DDM parameters and conflict-dependent neural responses were included to evaluate alternative explanations and provide complementary exploratory characterization.

In univariate activation analyses, we constructed a general linear model (GLM) identifying brain regions that respond parametrically to the task-relevant conflict at choice onset. We also included the task-irrelevant conflict and response time in the GLM as parametric modulators, thereby controlling statistically for their effects. Trial-by-trial response time is associated with the fluctuations of many psychological processes, such as domain-general response conflict (relating to motor response tendencies, but not specific information processing) [65], sustained attention [66], autonomic arousal [67], and conscious effort [68]. Modeling response time in the GLM thus helps us to dissociate these RT-related processes from task-relevant conflict, the decision variable of primary interest.

In the moral condition, we found positive parametric responses to the moral task-relevant conflict (RC_moral_) in the right posterior TPJ (rpTPJ), dorsolateral prefrontal cortex (DLPFC), and dorsomedial prefrontal cortex (DMPFC) (Fig 3A; S5 Table). That is, activation in these regions increased with the moral task-relevant conflict, even when controlling for task-irrelevant conflict and RT-related domain-general processes. Previous fMRI meta-analyses have shown that these brain areas are activated consistently during moral decision-making [69–71]. Our results suggest that these regions are not merely associated with moral choices in general, but are sensitive to trial-by-trial variation in moral conflict during decision-making.

**Fig 3:**
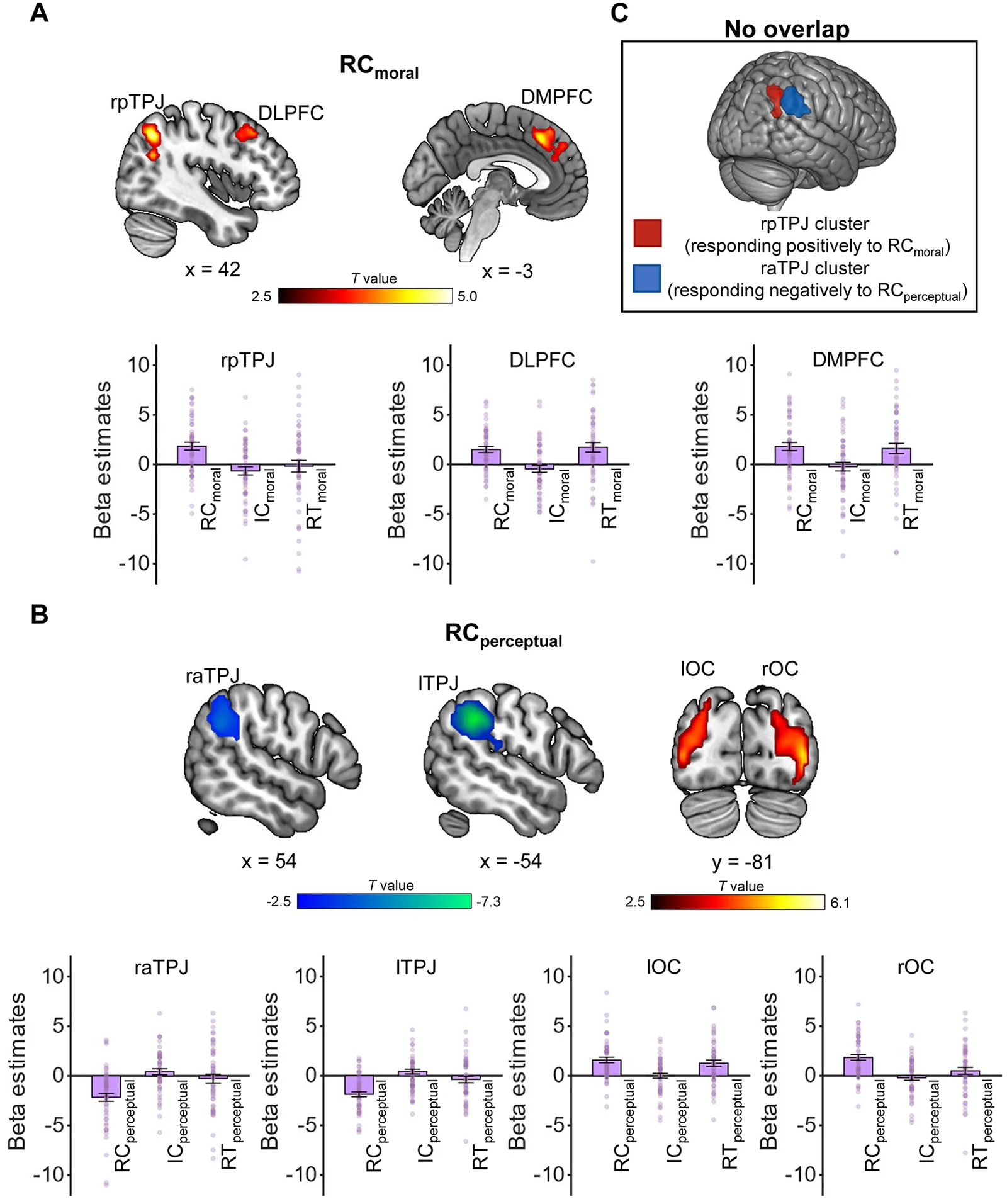
Moral and perceptual task-relevant conflict are represented by distinct brain areas. **(A, B)** Different brain regions were involved in representing moral task-relevant conflict (RC_moral_) and perceptual task-relevant conflict (RC_perceptual_). **(A)** Activity in right posterior temporoparietal junction (rpTPJ), dorsolateral prefrontal cortex (DLPFC), and dorsomedial prefrontal cortex (DMPFC) correlates positively with moral task-relevant conflict. **(B)** Activity in right anterior temporoparietal junction (raTPJ), left TPJ (lTPJ), left occipital cortex (lOC), and right occipital cortex (rOC) correlates negatively or positively with perceptual task-relevant conflict. Whole-brain FWE-cluster correction at *P* < 0.05 after cluster-forming threshold at *P* < 0.001. For illustrative purposes, we show the identified brain clusters’ mean beta estimates of the moral task-relevant conflict, task-irrelevant conflict, response time (RC_moral_, IC_moral_, and RT_moral_) and the mean beta estimates of perceptual ones (RC_perceptual_, IC_perceptual_, and RT_perceptual_). Error bars represent the standard errors. Each dot indicates the estimate of an individual participant. **(C)** The two right temporoparietal junction clusters (rpTPJ and raTPJ) identified in different analyses had no overlap. The underlying data for this figure are available in the “Data” folder of our OSF repository.

In the perceptual condition, we found a different activation pattern. Consistent with the literature [72–74], perceptual task-relevant conflict (RC_perceptual_) correlated parametrically with activity in several regions often found involved in perceptual choices: The right anterior TPJ (raTPJ) and left TPJ (lTPJ) had negative parametric responses to the perceptual task-relevant conflict, while the right and left occipital cortex (rOC and lOC) had positive parametric responses to it (Fig 3B). Importantly, the brain regions responding to moral task-relevant conflict were spatially separated from those responding to the perceptual task-relevant conflict (Fig 3A and 3B). Even though they were located next to each other, there was no overlap between the rpTPJ cluster responding to moral task-relevant conflict and the raTPJ cluster responding to perceptual task-relevant conflict (Fig 3C). Moreover, a conjunction analysis showed that no region responded significantly to both the moral and perceptual task-relevant conflict.

To further explore potential associations between neural processing of moral and perceptual task-relevant conflict, we tested whether individual brains that showed stronger parametric responses to the moral task-relevant conflict also showed stronger parametric responses to perceptual conflict, using region of interest (ROI) analysis. We selected the regions of interest as the seven clusters that showed significant responses across the whole group to either moral or perceptual task-relevant conflict (Fig 3A and 3B). This selection criterion ensured that the analysis used to select the ROIs (group averages) did not confound the orthogonal analysis of correlations between the activity levels across the two choice types. We extracted the beta estimates from the RC_moral_-related parametric contrast images for the rpTPJ, DLPFC, and DMPFC clusters and from the RC_perceptual_-related parametric contrast images for the raTPJ, lTPJ, and bilateral OC clusters and computed Pearson correlations between them. This revealed no significant correlations after false discovery rate correction (S6 Table), thereby providing no evidence, in this ROI analysis, for shared individual-level neural sensitivity to moral and perceptual conflict. This result is consistent with the spatial separation observed in the main activation analysis.

To directly test the neural specificity of moral and perceptual conflict processing, we compared the neural activity related to moral task-relevant conflict processing with the activity related to perceptual task-relevant conflict processing (i.e., RC_moral_ versus RC_perceptual_). The results showed that the activity in rpTPJ and DLPFC that positively correlated with the moral task-relevant conflict was more specific to moral than perceptual task-relevant conflict, as these two regions had significantly more positive parametric responses to moral task-relevant conflict than to perceptual task-relevant conflict (Fig 4A). However, the activity in the DMPFC that positively correlated with moral task-relevant conflict was not significantly different from the corresponding activity in response to perceptual task-relevant conflict. Moreover, this analysis showed that activity in the raTPJ, lTPJ, and bilateral OC was more specific to perceptual than moral task-relevant conflict: These regions’ parametric responses to the perceptual task-relevant conflict were significantly different from those to the moral task-relevant conflict (Fig 4A and 4B). Finally, we found that activity in the ventrolateral prefrontal cortex (VLPFC) had more positive parametric responses to moral than perceptual task-relevant conflict, while the inferior temporal cortex (ITC) had more positive parametric responses to the perceptual than moral task-relevant conflict.

**Fig 4:**
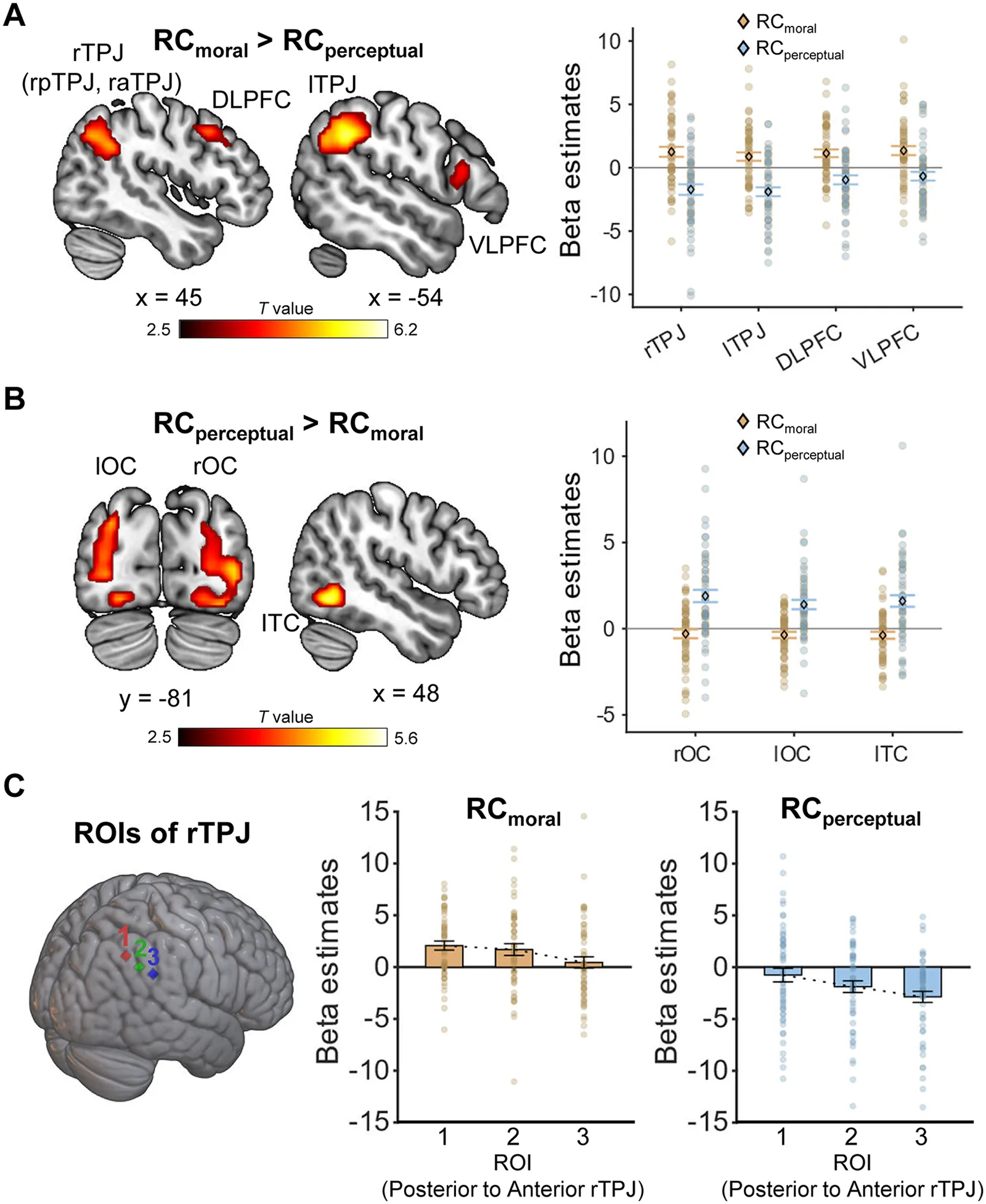
Neural specificity of moral and perceptual task-relevant conflict processing. **(A)** The bilateral temporoparietal junction (TPJ), dorsolateral prefrontal cortex (DLPFC), and ventrolateral prefrontal cortex (VLPFC) exhibited stronger positive parametric responses to the task-relevant conflict in the moral condition (RC_moral_) compared to that in the perceptual condition (RC_perceptual_). **(B)** The right and left occipital cortex (rOC and lOC) and inferior temporal cortex (ITC) exhibited stronger positive parametric responses to the task-relevant conflict in the perceptual condition compared to that in the moral condition. **(A, B)** Shown are mean beta estimates of the identified clusters in the moral and perceptual conditions. Error bars represent the standard errors. Each dot indicates the estimate of an individual participant. Whole-brain FWE-cluster correction at *P* < 0.05 after cluster-forming threshold at *P* < 0.001. r, right; l, left; a, anterior; p, posterior. **(C)** Spatial gradient for the moral and perceptual task-relevant conflict processing within the rTPJ. Three regions of interest (ROIs) were defined along the posterior to anterior rTPJ. In more anterior ROIs, the positive parametric responses to the moral task-relevant conflict became weaker, while the negative parametric responses to the perceptual task-relevant conflict became stronger. Dotted lines connect the mean values across different ROIs. Error bars represent the standard errors. These ROI estimates are shown for visualization only, and no statistical tests were conducted on them to avoid circular analysis. The underlying data for this figure are available in the “Data” folder of our OSF repository.

We also used an ROI analysis to test whether moral and perceptual task-relevant conflicts are encoded differentially in brain areas related to morality, as identified by a meta-analysis [70]. Consistent with our whole-brain analysis, the ROI analysis showed that the bilateral TPJ areas previously associated with morality [70] exhibited more positive parametric responses to moral task-relevant conflict than to perceptual task-relevant conflict (S6 Fig; S7 Table).

Intriguingly, our analyses thus showed that the right TPJ (rTPJ) responded to both moral and perceptual task-relevant conflicts, but in opposite ways (Fig 4A). This reflected that a posterior part of the rTPJ had positive responses to the moral task-relevant conflict, whereas an anterior part of the rTPJ had negative responses to the perceptual task-relevant conflict (Fig 3C). To visualize this posterior-to-anterior pattern, we created three regions of interest (ROIs) along the right posterior-to-anterior TPJ (Fig 4C). The ROIs were created as 3-mm spheres centered on the peaks of the rpTPJ and raTPJ clusters identified by the RC_moral_- and RC_perceptual_-related parametric contrasts in the univariate activation analysis and the midpoint between the two peaks. The MNI coordinates of the three ROIs were: [42, −60, 42], [51, −54, 36], and [60, −48, 33]. We extracted the beta estimates from the RC_moral_- and RC_perceptual_-related parametric contrast images for each ROI and visualized them. Avoiding circular analysis [75], we did not conduct any statistical analysis on these beta estimates. However, these visualizations illustrate that the positive parametric responses to the moral task-relevant conflict became progressively weaker in more anterior ROIs, while the negative parametric responses to the perceptual task-relevant conflict became progressively stronger in more anterior ROIs (Fig 4C).

### Control and supplementary analyses

We conducted several control and supplementary analyses to characterize control regressors, assess the robustness of the main task-relevant-conflict findings, examine possible nonlinear conflict effects, and evaluate value-related alternative explanations. Our GLM analysis (GLM 1) included task-irrelevant conflict and response time as trial-level parametric modulators, thereby statistically controlling for conflicts of no interest and domain-general processes related to response time, such as response conflict [65], sustained attention [66], and conscious effort [68]. To provide a complementary characterization of these control regressors, we examined the neural correlates of task-irrelevant conflict and response time. Consistent with previous findings that response time explains a substantial amount of variance in the neural activation across different tasks [66], our analysis showed that RT-related regions were largely similar in the moral and perceptual conditions (Fig 5A and 5B; S8 Table). A conjunction analysis revealed common RT-related activation in the middle cingulate cortex (MCC), bilateral LPFC, bilateral insula, precuneus, and occipital cortex (OC) (Fig 5C). No region revealed significant responses to task-irrelevant conflict in the moral (IC_moral_) or perceptual (IC_perceptual_) condition.

**Fig 5:**
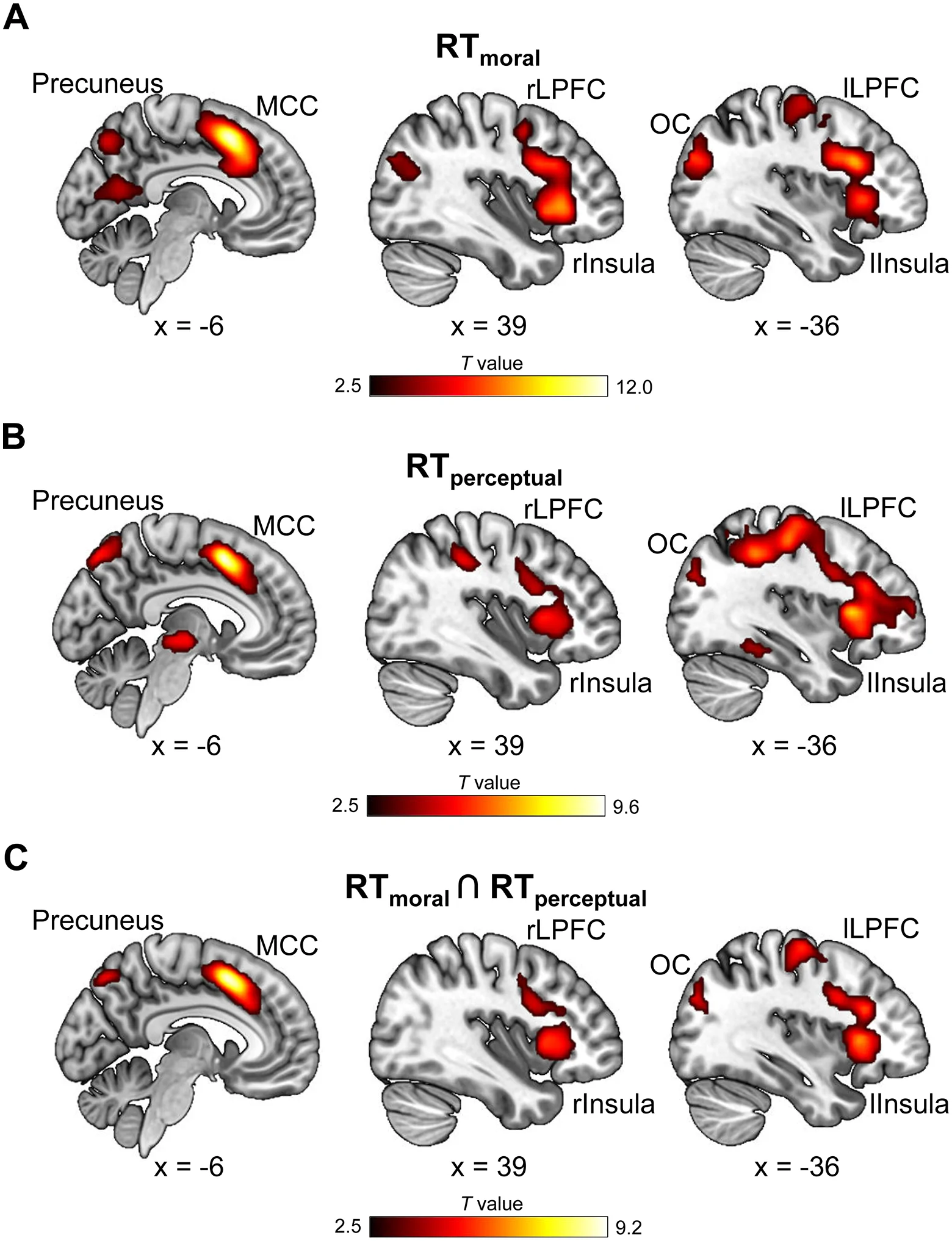
Similar response-time-related neural activity in moral and perceptual choices. **(A, B)** Similar brain regions showed activity correlated with response time in the moral (**A**) and perceptual (**B**) conditions (RT_moral_ and RT_perceptual_). **(C)** A conjunction analysis showed that common RT-related regions included the middle cingulate cortex (MCC), precuneus, bilateral lateral prefrontal cortex (LPFC), bilateral insula, and occipital cortex (OC). r, right; l, left. Whole-brain FWE-cluster correction at *P* < 0.05 after cluster-forming threshold at *P* < 0.001.

To assess the robustness of the main task-relevant-conflict findings, we conducted two additional control analyses. The results were similar regardless of whether task-relevant conflict, task-irrelevant conflict, and response time were included in the same GLM or in separate GLMs, and regardless of whether the analyses were restricted to correct trials or included all trials (see Materials and methods; S9, S10, S11, S12 Tables; S7 Fig).

As an additional supplementary analysis, we examined whether task-relevant conflict was related to brain activation in a nonlinear manner by including quadratic and cubic terms of conflict in the GLM (see Materials and methods). The caudate showed significant negative parametric responses to the quadratic term of moral conflict (*T* = 4.28, *P*_*FWE*_ = 0.004, peak MNI coordinates: [−6, 12, 15]; cluster size: 106 voxels); however, these responses did not significantly differ from those to the quadratic term of perceptual conflict. No region exhibited significant responses to the quadratic term of perceptual conflict or to the cubic terms of either moral or perceptual conflict. These supplementary analyses provided no evidence for domain-specific nonlinear task-relevant-conflict effects.

Unlike some previous studies showing that classical valuation regions, such as the VMPFC and striatum, are involved in moral decision-making [23], we did not observe robust engagement of these regions in our main analyses. One possibility is that classical valuation regions may encode chosen value rather than resolve decision conflict. To examine this possibility, we conducted an additional supplementary analysis of the neural correlates of chosen value (see Materials and methods). Chosen value was defined as the moral rating of the selected picture in the moral condition and as the area size of the selected picture in the perceptual condition. The results showed that neither moral nor perceptual chosen value elicited significant responses in classical valuation regions, such as the VMPFC or striatum (S13 Table). Although the conjunction analysis revealed overlapping responses to moral and perceptual chosen value, these overlaps were located in posterior visual regions rather than in canonical valuation regions. Thus, the absence of classical valuation regions in our main analyses is unlikely to simply reflect our focus on conflict-related signals rather than chosen-value signals.

### Distinct conflict-dependent functional connectivity during moral and perceptual choices

The neural implementation of choice mechanisms can be specialized not only in terms of regional activation but also in terms of functional connectivity between brain regions [20,21]. It is therefore possible that moral conflict processing relies not only on domain-specific brain activation but also on domain-specific functional connectivity. We therefore extended our analysis to the circuit level by conducting psychophysiological interaction analyses to identify brain-region interactions that covaried with the strength of moral or perceptual task-relevant conflict. As seed regions for these analyses, we used the clusters that responded parametrically to moral or perceptual task-relevant conflict in the univariate activation analyses (Fig 3A and 3B; see Materials and methods). These analyses revealed that two regions involved in moral task-relevant conflict processing (rpTPJ and DMPFC; Fig 3A) showed moral-conflict-dependent modulations of functional connectivity with visual areas and the precuneus. Specifically, functional connectivity between rpTPJ–rOC, rpTPJ–lOC, DMPFC–lOC, and DMPFC–precuneus increased as moral task-relevant conflict became stronger (Fig 6A; S14 Table). By contrast, no functional connectivity patterns were significantly correlated with perceptual task-relevant conflict, either positively or negatively.

**Fig 6:**
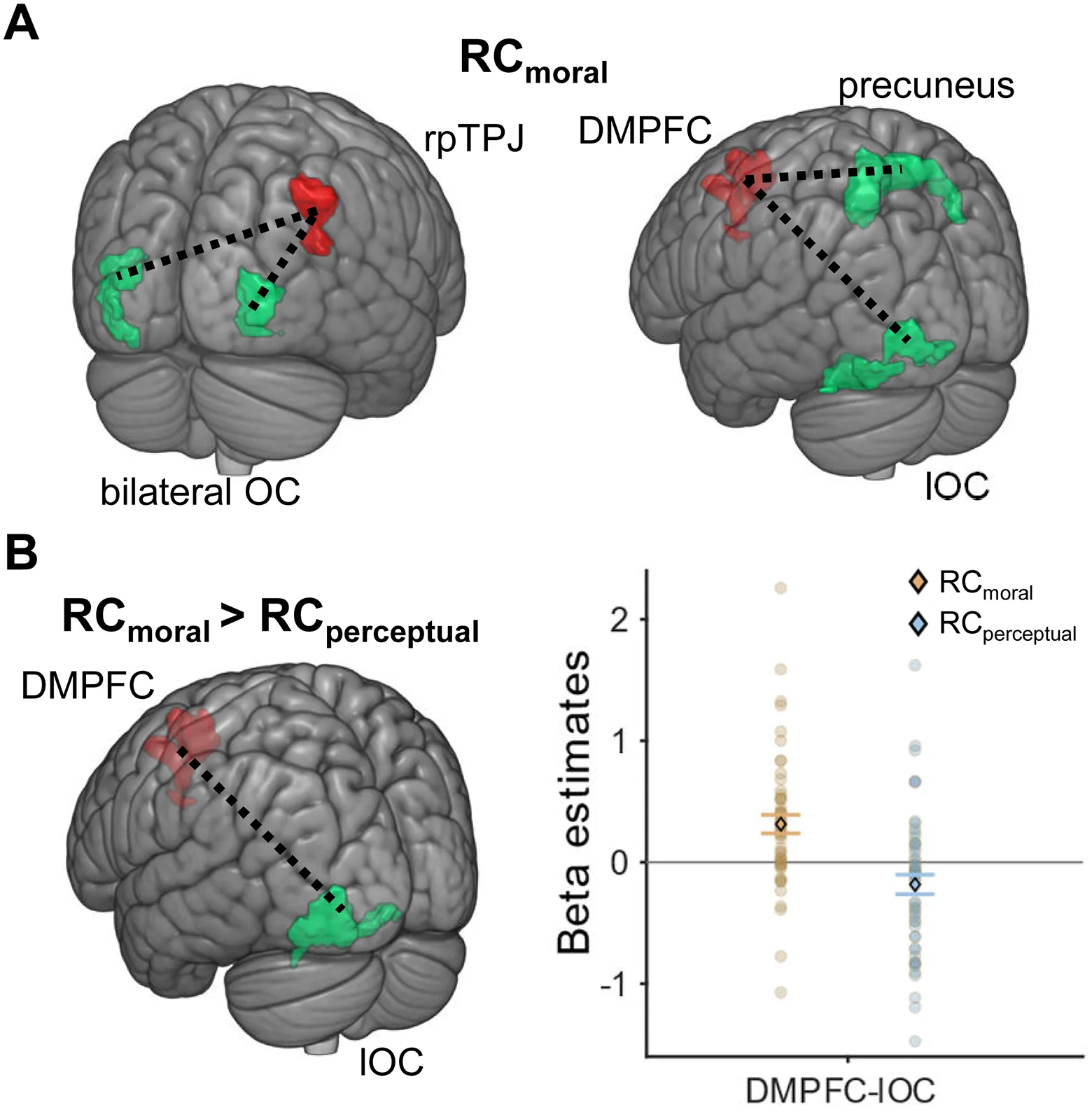
Functional connectivity related to moral task-relevant conflict. **(A)** Single-condition psychophysiological interaction (PPI) analyses showed that higher moral task-relevant conflict (RC_moral_) was associated with more positive functional connectivity between the right posterior temporoparietal junction (rpTPJ) and bilateral occipital cortex (OC), as well as between the dorsomedial prefrontal cortex (DMPFC) and left OC and precuneus. **(B)** The DMPFC–lOC connectivity showed a more positive association with the task-relevant conflict in the moral condition than in the perceptual condition (RC_perceptual_), reflecting the condition-relative contrast RC_moral_ > RC_perceptual_. Shown are mean beta estimates of the functional connectivity in the moral and perceptual conditions. Error bars represent standard errors. Each dot indicates the estimate of an individual participant. Seed regions are presented in red and identified clusters are presented in green. Whole-brain FWE-cluster correction at *P* < 0.05 after cluster-forming threshold at *P* < 0.001. r, right; l, left. The underlying data for this figure are available in the “Data” folder of our OSF repository.

To further test whether the identified functional connectivity modulations were more specific to moral conflict, we conducted direct contrasts (RC_moral_ versus RC_perceptual_). Only DMPFC–lOC connectivity showed evidence of domain specificity, with a stronger positive association with moral than perceptual task-relevant conflict (Fig 6B). These findings suggest that moral conflict processing was associated with conflict-dependent functional connectivity patterns, particularly involving DMPFC–lOC coupling. Thus, in addition to domain-specific regional activation, moral and perceptual conflict processing also differed in some aspects of circuit-level implementation.

### Exploratory domain-specific neural associations with drift-diffusion model parameters

Drift-diffusion model parameters characterize individual differences in choice processes at the algorithmic level. Among them, the drift-rate scale and threshold are closely related to conflict processing and were therefore our primary parameters of interest. Examining their neural correlates allowed us to test whether algorithmic individual differences related to moral and perceptual conflict processing showed domain-specific associations with conflict-dependent neural responses. We included the individual drift-rate scale and threshold parameters of moral and perceptual choices as second-level covariates, building on the univariate activation and psychophysiological interaction analyses described above.

For regional activation, parametric responses to perceptual task-relevant conflict in the ITC were positively associated with the perceptual threshold (Fig 7A; S15 Table). This association showed domain specificity, as ITC activation was more positively associated with the perceptual threshold than with the moral threshold (Fig 7B; S15 Table). By contrast, no regional activation responses to moral task-relevant conflict were significantly associated with moral DDM parameters. These exploratory findings provide evidence for a perceptual-domain-specific link between regional activation and choice caution.

**Fig 7:**
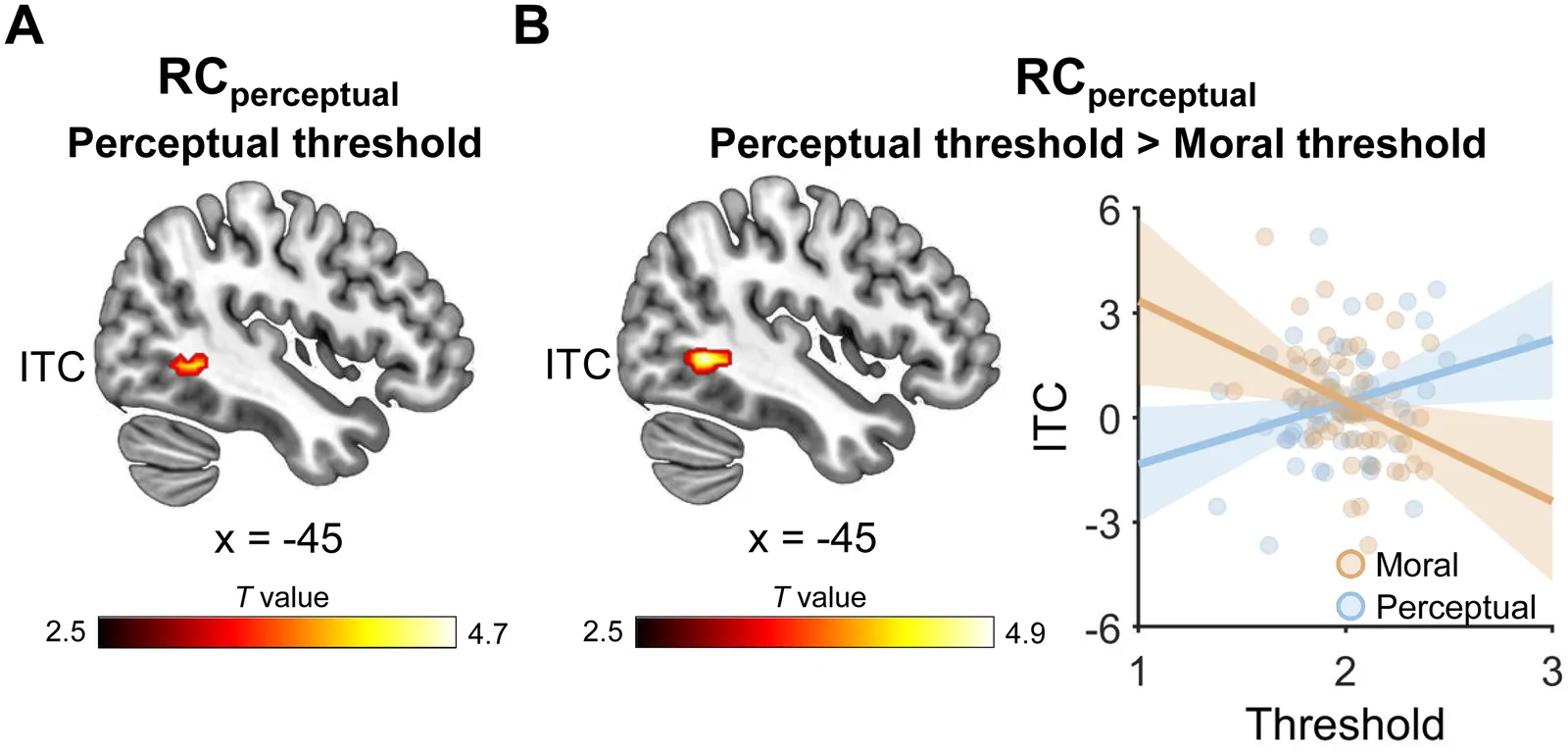
Brain activation associated with perceptual threshold. **(A)** Exploratory individual-difference analyses examined whether individual differences in drift-rate scale and threshold parameters were associated with perceptual-conflict-related regional activation estimated from the single-condition parametric modulation analysis of perceptual task-relevant conflict (RC_perceptual_). Larger perceptual thresholds were associated with more positive perceptual-conflict-related activation in the inferior temporal cortex (ITC). **(B)** Direct comparison tested which brain regions were more strongly associated with the perceptual threshold than with the moral threshold. The ITC activation as RC_perceptual_ increased showed a more positive association with the perceptual threshold than with the moral threshold. Whole-brain FWE-cluster correction at *P* < 0.05 after cluster-forming threshold at *P* < 0.001. Scatter plots are shown solely for illustrative purposes. Each line represents the least squares fit with shading showing the 95% confidence interval. Each dot indicates the estimate of an individual participant.

For functional connectivity, in the moral condition, moral-conflict-dependent rpTPJ–MCC and rpTPJ–superior parietal cortex (SPC) connectivity were positively associated with the moral drift-rate scale and threshold, respectively (Fig 8A; S16 Table). In the perceptual condition, perceptual-conflict-dependent connectivity between lTPJ–cuneus was positively associated with the perceptual threshold. We then directly compared whether each connectivity effect was more strongly associated with the corresponding DDM parameter from the same choice domain than with that from the other choice domain. Two moral-conflict-dependent connectivity effects showed such domain-specific associations with moral DDM parameters: rpTPJ–MCC connectivity showed a stronger positive association with the moral drift-rate scale than with the perceptual drift-rate scale, and rpTPJ–SPC connectivity showed a stronger positive association with the moral threshold than with the perceptual threshold (Fig 8B and 8C; S16 Table). These exploratory findings provide evidence for moral-domain-specific links between conflict-dependent functional connectivity and algorithmic components of choice, particularly information-processing efficiency and choice caution.

**Fig 8:**
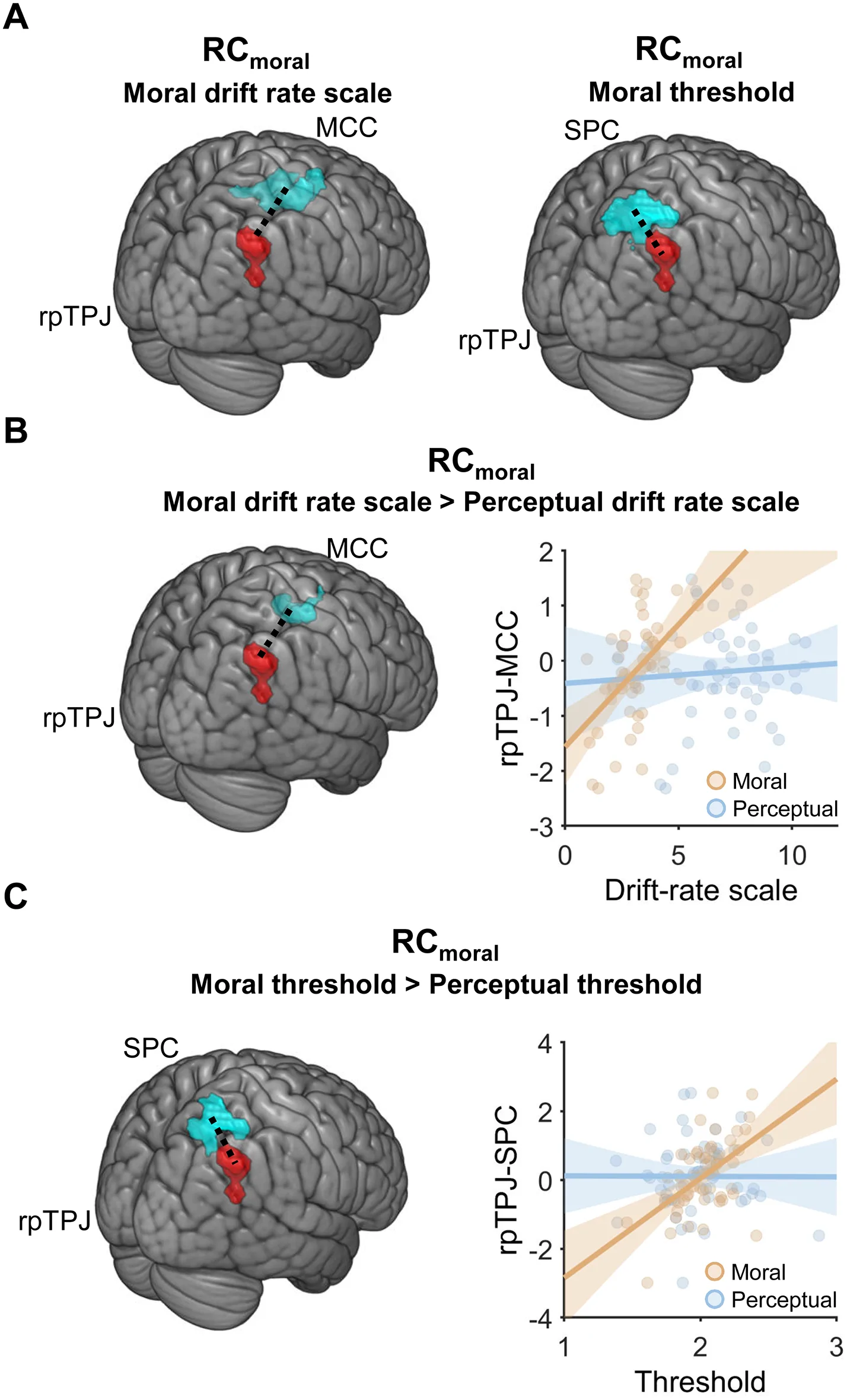
Functional connectivity associated with moral drift-rate scale and threshold. **(A)** Exploratory individual-difference analyses examined whether individual differences in drift-rate scale and threshold parameters were associated with moral-conflict-dependent functional connectivity estimated from the single-condition psychophysiological interaction (PPI) analysis of moral task-relevant conflict (RC_moral_). Larger moral drift-rate scales were associated with more positive moral-conflict-dependent rpTPJ–MCC connectivity. Larger moral thresholds were associated with more positive moral-conflict-dependent rpTPJ–SPC connectivity. **(B, C)** Direct comparisons tested which moral-conflict-dependent connectivity effects were more strongly associated with moral than perceptual DDM parameters of the same type. The rpTPJ–MCC connectivity as RC_moral_ increased showed a more positive association with the moral drift-rate scale than with the perceptual drift-rate scale **(B)**, and the rpTPJ–SPC connectivity as RC_moral_ increased showed a more positive association with the moral threshold than with the perceptual threshold **(C)**. Seed regions are presented in red and identified clusters are presented in blue. Whole-brain FWE-cluster correction at P < 0.05 after cluster-forming threshold at *P* < 0.001. Scatter plots are shown solely for illustrative purposes. Each line represents the least squares fit with shading showing the 95% confidence interval. Each dot indicates the estimate of an individual participant.

## Discussion

A long-standing question in both psychology and neuroscience is whether moral decision-making is special—is it similar to other, more mundane types of choices and therefore governed by domain-general neurocognitive processes, or is it a unique human ability that is governed by domain-specific neurocognitive processes that constitute their own choice apparatus [9–13]. The neural common currency hypothesis [19,21], and moral metaphor studies [25,26] emphasize the similarity between moral and non-moral (i.e., perceptual) choices, whereas the social brain hypothesis [27,28] suggests that moral choices are fundamentally distinct. Here, we empirically examined this long-standing question.

To this end, we considered different levels for the analysis of information processing systems, as proposed by Marr [58], and systematically examined the neural differences and commonalities between moral and non-moral choices. At the computational level, our new experimental paradigm ascertained that moral and non-moral choices were separable, since our participants were required to make moral or perceptual choices (with different goals) on the same set of pictures, using an identical form of motor outputs. At the algorithmic level, we then examined whether these matched moral and non-moral choices were governed by the same decision processes. As the literature has documented the influence of conflict levels on choices in both moral and perceptual domains, and as the related decision processes can be captured by the drift-diffusion model [60,61], we manipulated the conflict levels of moral and perceptual choices in our task in a manner that fully separated these two decision variables between the two types of choices. Participants’ moral and perceptual choices were specifically affected only by the task-relevant conflict, but modeling showed that the two different types of information (moral and perceptual) were processed by a similar choice mechanism that was captured by the same DDM. Even though moral and perceptual choices were similar algorithmically, analysis at the implementation level showed considerable differences. That is, fMRI analysis showed that moral conflict was represented by domain-specific neural activity that differed from that involved in perceptual conflict processing, in terms of involved brain regions, directions of correlations between BOLD activity and conflict, and patterns of functional connectivity. Importantly, statistical control analyses ascertained that the neural responses to moral conflict we identified are clearly separate from domain-general basic psychophysiological processes that vary with trial-by-trial response time (e.g., response conflict, sustained attention, and conscious effort). Thus, our findings provide evidence for distinct neural processes underlying conflict resolution in moral relative to perceptual choices, consistent with the social brain hypothesis.

### Neural correlates of moral conflict

Our findings that moral conflict was represented in the rpTPJ and DLPFC are generally consistent with the results of fMRI meta-analyses showing that these brain regions are related to moral cognition [69–71]. More specifically, Morishima and colleagues [76] found that the right TPJ was most active for donation choices that involved a monetary cost that just matched individuals’ willingness to pay, thus entailing maximum conflict between self-interest and moral considerations. Obeso and colleagues [52] used transcranial magnetic stimulation (TMS) to show that the right TPJ was causally involved in resolving the monetary-moral conflict related to altruistic choices. As for the right DLPFC, Knoch and colleagues [77] found that TMS of this region biased the monetary-moral conflict associated with fairness, and Maréchal and colleagues [78] showed with transcranial direct current stimulation (tDCS) that stimulating DLPFC affected the resolution of monetary-moral conflict. Of note, while the prior studies have linked the activity of the right TPJ and DLPFC to the conflict between obeying moral norms and gaining monetary reward, it remains an open question whether moral choices without any financial consequences engage these regions. Our findings thus extend previous studies by revealing that moral conflict in choices based on violation severity is tracked by the right posterior TPJ and DLPFC.

We also found that the DMPFC represented moral conflict. Even though no previous study has directly indicated the DMPFC’s involvement in moral conflict processing, meta-analyses have consistently associated its activation with moral cognition in general [70,71], and studies have often found it to be implicated in understanding others’ mental states (i.e., theory-of-mind processing) during moral tasks [79,80]. Our results suggest a more specific function of this region for moral decision-making, namely comparing and processing the conflict between decision options when the relevant decision variable is the difference in their moral value.

In contrast to some previous studies [23], we did not observe robust engagement of classical valuation regions (e.g., VMPFC, striatum) during moral choices. This discrepancy may be due to differences in choice type and task design: In many previous studies [22,23,81], moral decisions involved explicit trade-offs between obeying moral norms and gaining monetary rewards, which may have required participants to integrate different types of value onto a common scale for comparison, thereby potentially recruiting classical valuation regions associated with domain-general value integration [19]. By contrast, our task required participants to compare the severity of moral violations without direct financial consequences, thus not containing explicit trade-offs between moral and non-moral values. These findings suggest that classical valuation regions may not necessarily be engaged during moral information processing in such contexts.

Alongside localized brain regions, brain-wide circuits responded to moral conflict as well. We found that functional connectivity of TPJ and DMPFC with occipital regions and precuneus increased with moral conflict. Different from the high-level regions integrating information from multiple sources (e.g., TPJ and DMPFC), the bilateral occipital cortices are low-level sensory regions sensitive to basic visual properties, including color [82], shape [83,84], size [85,86] and so on. The coupling of the rpTPJ and DMPFC with these regions might indicate visual information propagation from low-level sensory regions to high-level integrative regions [87] and/or the top-down control of sensory information processing of low-level regions by high-level regions [88,89]. This might relate to the need for deeper visual processing to understand the intention and behavior of the agents when judging the morality of depicted actions, in particular when this morality is more similar between the choice options. The precuneus has been implicated in autobiographical memory [90]; its functional coupling with DMPFC might therefore reflect individuals’ effort in extracting useful information and knowledge from their memory for representing and solving the moral conflict. These results suggest that the rpTPJ and DMPFC may have to draw on fine-grained information presented in the pictures and from their memory to resolve moral information (e.g., the intentions of agents).

### Neural correlates of perceptual conflict

Our results that perceptual conflict was represented in the raTPJ, left TPJ, and bilateral OC are in line with previous findings. It has been found that activation of the TPJ increases with the perceptual evidence (i.e., the complement of the conflict) [72,74], suggesting the TPJ’s role in reflecting task-relevant salience in the perceptual domain [91,92]. The OC is known to be involved in object size perception [86,93,94]. No functional connectivity significantly changed in strength with perceptual conflict. Thus, unlike the finding of conflict-related rpTPJ–OC coupling during moral choices, the raTPJ did not appear to interact functionally with the OC via functional connectivity during perceptual choices. These different functional connectivity patterns imply that the rpTPJ and raTPJ may have different functions related to morality and basic perception, as discussed in more detail in the following paragraphs.

### Distinct neural implementations of moral and perceptual conflict

We found that the representation of moral and perceptual conflict was subserved by distinct brain regions and circuits (Figs 3A, 3B, and 6A). In addition, direct comparisons between the neural activity evoked by moral and perceptual conflict further demonstrated that these responses were specific for one type of decision conflict (Figs 4A, 4B, and 6B). Crucially, the activation of TPJ and DLPFC to moral conflict, and the moral-conflict-related connectivity between DMPFC and lOC, were statistically stronger for moral than perceptual conflicts (Figs 4A and 6B). These findings show that the brain comprises a domain-specific system that is more sensitive to moral conflict than perceptual conflict, thereby supporting the neural specificity of moral choices.

The TPJ has often been linked with a range of psychological functions (see a review [95]), and it is debated whether the TPJ should be seen as a unitary processing unit with an overarching domain-general function or as a set of functionally specialized subregions (e.g., [96,97]). Our results provide evidence for the latter view. We found that the right TPJ, considered as a whole, responded positively to increased moral conflict but negatively to increased perceptual conflict (Fig 4A), suggesting different functional contributions to these two domains of behavioral control. The positive response to moral conflict is consistent with previous findings that the TPJ is more engaged when moral conflict is high (e.g., [76]). This pattern may reflect the increasing demands of mental simulation, perspective-taking, and social meaning construction when the relative severity of two moral violations is difficult to compare. By contrast, the negative response to perceptual conflict is consistent with the suggestion that the TPJ is sensitive to task-relevant salience or attentional capture [91,92]. In perceptual trials, lower conflict corresponds to larger differences in area size between the two options, making one option more salient and easier to select. Thus, stronger TPJ responses under low perceptual conflict may reflect attentional capture by more salient perceptual evidence. These opposing response profiles suggest distinct underlying computational principles within the TPJ: cognitive simulation for moral conflict versus attentional capture by perceptual evidence strength.

This functional distinction was further supported by the spatial separation of the TPJ clusters. Specifically, there was no overlap between the posterior TPJ cluster responding positively to moral conflict and the anterior TPJ cluster responding negatively to perceptual conflict (Fig 3C). Studies using cytoarchitectonic, tractography-based, and functional parcellation have indicated that the TPJ can be fractionated into at least two subdivisions: the anterior and posterior TPJ [95,97,98]. The anterior TPJ has been more strongly associated with non-social processes (e.g., attention reorientation), whereas the posterior TPJ has been more strongly associated with social processes (e.g., theory of mind) [96,97,99–101]. Consistent with this subdivision, the right TPJ’s positive parametric response to moral conflict became weaker from posterior to anterior regions, whereas its negative parametric response to perceptual conflict became stronger from posterior to anterior regions (Fig 4C). Together, these findings reveal differential spatial gradients within the TPJ for domain-dependent conflict processing, suggesting that anterior and posterior TPJ subregions contain neurons with distinct computational functions rather than neurons implementing a single domain-general conflict-processing mechanism.

Apart from conflict representation, the neural implementation of individual differences in the algorithmic components of choice processes also appears to be domain-specific. Despite their significant correlations, moral and perceptual DDM parameters were associated with distinct neural patterns (S15 and S16 Tables). Notably, direct comparisons revealed that moral-conflict-dependent modulations of rpTPJ–MCC and rpTPJ–SPC connectivity were more strongly correlated with the moral drift rate scale and threshold, respectively, than with their perceptual counterparts (Fig 8B and 8C). The MCC is a key node within the cognitive control network, implicated in the dynamic allocation of attention according to task demands [102,103]. Participants with higher moral information processing speed, as reflected by the drift rate parameter, may benefit from more efficient allocation of attentional resources. Specifically, when moral conflict is high, the MCC may recruit greater attentional resources to support the rpTPJ in resolving the conflict, whereas under low-conflict conditions, attentional engagement may be reduced to conserve cognitive resources. The superior parietal cortex (SPC) is involved in sustaining goal-directed attention [104,105]. Participants with higher choice caution, as indicated by the threshold parameter, may rely more heavily on sustained attention to maintain elevated decision thresholds and complete the required accumulation of evidence. Specifically, under conditions of high moral conflict, maintaining high decision thresholds may require increased engagement of the SPC to provide sustained attentional support to the rpTPJ, thereby facilitating the full representation of available evidence and the resolution of conflict.

In contrast to the moral domain, where domain-specific neural associations of algorithmic choice components were expressed through inter-regional coordination, the perceptual domain showed a domain-specific association between the threshold parameter and regional activation. Specifically, ITC activation was more positively associated with the perceptual threshold than with the corresponding moral threshold (Fig 7B). Given the ITC’s role in higher-level visual processing, including object-form analysis and visual feature integration [106,107], this finding suggests that participants with greater perceptual choice caution may recruit the ITC more strongly under high-conflict perceptual conditions, possibly reflecting greater demands on the sustained processing or maintenance of perceptual task-relevant evidence.

An interesting question is why the brain may comprise a specialized neural system for moral information processing. According to the social brain hypothesis, this may be because selection pressures from complex social interactions may have uniquely shaped our brain during evolution. Compared with living alone, living in social groups is more cognitively demanding, as individuals must be able to coordinate their cognition and behavior with other group members in order to protect themselves from punishment and to maintain group cohesion [30]. Viewed from this angle, moral conflicts as a prime example of social information relevant for social interactions may thus engage specific neural circuits shaped by evolutionary pressures to cooperate and comply with norms governing life in social groups. Although the social brain hypothesis has gathered support from several neuroimaging studies, these mainly focused on other aspects of social interactions rather than moral conflicts as examined here (for reviews, see [20,21]).

### Neural correlates of response time

Consistent with previous studies [66,108], we found that response time correlated with activation of several brain regions. These regions, especially the MPFC (including MCC) and LPFC, are key nodes of a cognitive control network involved in monitoring behavioral performance, providing sustained attention, and supporting goal-directed behavior [102,109–111]. We also found that the RT-related regions were largely similar during the moral and perceptual choices. Thus, our findings indicate that these regions are responsible for basic psychophysiological processes important for response selection during all types of choices, but are not dedicated to processing domain-specific information (e.g., moral and perceptual conflicts). Echoing this account, the correlations between the activation of the MCC, MPFC, LPFC and RT have been found in various types of tasks, including a simple button-press task [108], a memory task [66], and a moral task [112].

Importantly, response time is closely linked to DDM parameters, as these parameters are derived from both choice and response-time distributions. Thus, the largely overlapping RT-related brain networks observed during moral and perceptual choices may partly reflect the neural implementation of their shared algorithmic structure. In this sense, the RT-related neural findings complement our behavioral DDM results by suggesting a potential neural basis for the algorithmic similarity between moral and perceptual choices.

### Moral objectivity as a psychological experience

Moral objectivism is a metaethical view holding that at least some moral claims are objective and factual, such that they can be true or false independently of individual beliefs [113–117]. Inspired by this philosophical view, psychological research has examined people’s intuitions concerning moral objectivity, that is, the extent to which moral information is perceived as fact-like rather than preference-like [118,119]. Previous studies have shown that people sometimes regard moral information as objective in specific contexts [120–126]. Related neuroscientific work on naïve realism and subjective construal further suggests that socially constructed meanings and attitudes can be experienced as objective features of the world [127,128]. Our findings may help explain this experience: the algorithmic similarity between moral and perceptual choices, together with overlapping response-time-related neural activity across tasks, may contribute to the subjective impression that moral and objective non-moral (i.e., perceptual) information should be considered in similar ways during decision-making. This provides one possible psychological explanation for why moral information may sometimes be experienced as objective-like or fact-like. Future studies could further evaluate this explanation by using standardized psychological scales to measure individual differences in intuitions concerning moral objectivity along a continuum [129], and by examining how these intuitions modulate the neurocomputational mechanisms underlying the processing of moral and objective non-moral information.

### Limitations and prospects

Moral processing may differ from other forms of non-moral processing along different task features. For example, moral processing and perceptual processing may differ in abstraction level, with moral choices involving more abstract social reasoning and perceptual choices relying more on concrete visual processing. However, our analyses identified conflict-dependent neural responses within each task domain before comparing these responses across domains, thereby reducing the influence of global differences in abstraction level between the moral and perceptual conditions (S8 Fig). Moreover, the regions exhibiting domain-specific conflict-related responses in the present study were largely distinct from those identified in prior meta-analytic work on abstract versus concrete concept processing [130]. Thus, the neural dissociations observed here are unlikely to be fully attributable to differences in abstraction level alone. Future studies comparing moral processing with various forms of non-moral processing, such as basic perception, aesthetic judgment, or taste-related evaluation, should specify and quantify the features that distinguish these domains from moral processing and examine how these features contribute to neural differences between moral and non-moral processing.

It is noteworthy that choices are guided by processes that unfold dynamically over time [131–135]. Our fMRI study demonstrates domain-specific neural representations of moral evidence, defined as the complement of moral conflict. However, given the relatively low temporal resolution of fMRI, the present analyses cannot determine when during the choice process these representations emerge or how they evolve during decision-making. Future studies should use techniques with higher temporal resolution, such as electroencephalography (EEG) or magnetoencephalography (MEG), to examine the temporal dynamics of moral and perceptual choices.

Advanced analytic approaches could further advance this line of research. To examine whether additional shared mechanisms may be detectable at the multivariate level, future studies could use multivariate pattern analysis (MVPA), including approaches based on parametric contrast maps, to further investigate the similarities and differences between moral and perceptual conflict processing. In addition, future studies could use brain-informed DDMs to examine whether trial-level neural activity in domain-specific regions or circuits predicts DDM parameters, thereby providing a more direct test of the relationship between algorithmic similarity and implementational specificity in moral and perceptual choices.

Decision-making is associated with multiple psychological processes. In addition to conflict resolution, decision-making also involves value construction and metacognitive confidence evaluation. Recent computational work has distinguished value or attribute construction from conflict resolution, the latter of which can be modeled as evidence accumulation, showing that these processes can have distinct neural dynamics and interpretations [136,137]. In the moral domain, value construction may involve integrating information about intention, outcome, causality, and responsibility into an overall evaluation of an option. Metacognitive confidence evaluation, by contrast, concerns judgments about the accuracy or reliability of one’s choices [138,139]. The present study focused on conflict resolution after the task-relevant dimension had been specified, rather than on value construction or confidence evaluation. Future studies could extend this broader perspective by examining how moral and non-moral choices differ or overlap in the neurocomputational mechanisms underlying value construction, conflict resolution, and metacognitive confidence evaluation.

Last but not least, our study only presented participants with morally blameworthy or neutral behaviors. Prior research suggests that moral blame and moral praise may have distinct mechanisms [140]. Future studies incorporating morally praiseworthy behaviors would be interesting for understanding whether similar mechanisms may resolve decision conflicts in the domains of positive moral behaviors.

In conclusion, our data indicate that even though the computational processes underlying moral and perceptual conflict processing are similar from an algorithmic point of view, they are implemented by distinct patterns of brain activation and functional connectivity. Our findings thus support the social brain hypothesis and provide evidence for the neural specificity of moral conflict processing.

## Materials and methods

### Participants

We recruited 53 healthy right-handed volunteers (students at University of Zurich and Federal Institute of Technology Zurich, mean age = 23.09 years, *SD* = 3.03, 26 females, 27 males). The sample size was based on a power calculation to have >80% power to detect medium to large correlations (*ρ* = 0.4) between variables across participants at *α* = 0.05. This sample size was not smaller than that in other moral studies using fMRI [22,53]. All participants had normal vision and reported no history of psychological or neurological disorder. They provided written consent and received monetary compensation for their participation. The participants were fully aware of the contents of the pictures they would see before they decided to participate in the experiment and did not report any problem about seeing the pictures after the experiment. The experimental protocol was approved by the Ethics Committee of the Canton Zurich (sub-study 41 under 2010-0327/3) and conformed to the Declaration of Helsinki.

### Personality scales

Participants were required to complete the Interpersonal Reactivity Index Scale [141] and Levenson Self-Report Psychopathy Scale [142] online at least one day before the experiment. These data are not reported in the current study.

### Stimuli

Our aim was to construct different levels of independent moral and perceptual conflict using the same set of social pictures. For achieving it, we needed to (1) have pictures containing both variable moral and perceptual information and (2) make these two types of information uncorrelated. To manipulate the moral information (i.e., moral wrongness), we selected pictures depicting various social situations, including gun violence, domestic abuse, robbery, alcohol use, sports competition, and so on. To avoid causing psychological trauma, the contents of the pictures were selected to not be more extreme than those shown during night-time TV programs devoted to criminal topics. Ninety-eight pictures that met the criteria were bought from a website (https://www.shutterstock.com/) and could be used for non-commercial activities. To manipulate the perceptual information (i.e., area size), we edited the pictures by covering some non-critical parts with gray patches. To decorrelate moral and perceptual information, we removed some pictures and slightly changed the picture sizes based on the participants’ average ratings of moral wrongness and area size on the pictures in seven pilot experiments. Seventy-three pictures (size of each picture: 1,024 × 768 pixels) were kept and used in the current study.

### Behavioral paradigm

The experiment comprised two main phases: (1) the rating task (without fMRI scanning), and (2) the choice task (with fMRI scanning). During the rating task, according to different questions, participants rated (rating scale: 0% to 100% in steps of 4%) the same set of 73 pictures. These pictures depicted various situations in which people interacted with one another, animals, or objects. For moral ratings, participants indicated “how severe is the moral violation depicted by the picture” (0% = it was not morally wrong at all, 100% = one of the most severe moral violations among all the pictures); for perceptual ratings, participants indicated “how much in percent does the picture cover the gray background within the white frame” (0% = not at all, 100% = completely) (S1 Fig). The moral and perceptual ratings were used to generate trials with different conflict levels for the choice task later. For an effective use of the moral scale, participants briefly viewed all the pictures before providing ratings. In addition, participants also indicated “how difficult is it to understand what is happening in the picture” (difficulty ratings) and “how aroused do you feel while viewing the picture” (arousal ratings) (see S17 Table for descriptive statistics). These additional rounds of rating increased participants’ familiarity with the pictures and enabled them to make quick decisions (within 3 s) in the following choice task.

After the rating task, for the following choice task, an algorithm selected a set of picture pairs divided into four different conflict levels based on the moral or perceptual ratings provided by each participant in the rating task. Picture pairs for moral choices were selected based on individual moral ratings. They had four conflict levels (1, 2, 3, 4) corresponding to the following difference values:


$$\begin{array}{c} |m_{\text{1}}\;-\;m_{\text{2}}|\;\in \;[\text{2}\text{8}\%,\;\text{2}\text{0}\%,\;\text{1}\text{2}\%,\;\text{4}\%], \end{array}$$

where *m* represents the moral rating of a picture, and |*m*_1_ − *m*_2_| represents the absolute value of the moral rating difference between the two pictures for a given trial. This relates inversely to the degree of moral conflict, with larger differences corresponding to smaller conflict. Since greater rating differences imply lower conflict, we defined moral conflict as 100% minus the absolute difference (with 100% representing the maximum possible rating on the scale) (S5 Fig). Picture pairs for perceptual choices were selected correspondingly based on individual perceptual ratings. They had four conflict levels (1, 2, 3, 4) corresponding to the following difference values:


$$\begin{array}{c} |p_{\text{1}}\;-\;p_{\text{2}}|\;\in \;[\text{1}\text{6}\%,\;\text{1}\text{2}\%,\;\text{8}\%,\;\text{4}\%], \end{array}$$

where *p* represents the perceptual rating of a picture, and |*p*_1_ − *p*_2_| represents the absolute value of the rating difference between two pictures. Following the same transformation used for moral conflict, perceptual conflict was defined analogously.

To match conflict levels between conditions, we selected the precise difference values for the moral (|*m*_1_ − *m*_2_| ∈ [28%, 20%, 12%, 4%]) and perceptual (|*p*_1_ − *p*_2_| ∈ [16%, 12%, 8%, 4%]) trials based on the results of initial pilot studies. Despite this calibration, participants in the final study had slightly higher choice accuracies and shorter response times in the perceptual than moral condition (Fig 2B, 2C, 2E, and 2F). Nevertheless, these small differences cannot confound our neural findings, as we controlled for the effects of response time and choice accuracy in the fMRI analysis (see further details in the following paragraphs and additional discussion in the Discussion section).

Afterwards, participants proceeded to perform the choice task. In the choice task, a pair of pictures was presented in each trial (Fig 1A). Participants chose one of the two pictures according to the condition they were in. In the moral condition, participants made moral choices by indicating “which picture depicts a more severe moral violation”, while in the perceptual condition, participants made perceptual choices by indicating “which picture covered more gray background within the white frame”. Trials started with the central presentation of a green letter (for 3 s to 7 s, jitter) indicating whether participants were in a moral (letter ‘M’, 0.2°) or perceptual trial (letter ‘A’, 0.2°). The letter remained on the screen throughout the trial to remind participants of the current trial type. At the same time, two white frames (length: 4.9°, width: 3.6°) surrounding gray backgrounds appeared above and below the letter. Then, the green letter turned to gray (for 1 s) suggesting pictures would be displayed soon. Afterwards, two pictures were presented within the two white frames, respectively. Participants had 3 s to indicate their choice by pressing one of two buttons on a response box with their right middle finger (lower picture) or their right index finger (upper picture). If participants made their choice in time, the white frame surrounding the chosen picture turned to red; otherwise, a feedback message was displayed reminding participants to respond more quickly and the trial was regarded as a missing trial. The choice task contained 320 trials divided into 10 sessions of 32 trials. Each session had 4 blocks, 2 moral blocks and 2 perceptual blocks. Each block consisted of the same type of trials, the number of which was pseudo-randomized to between 7 and 9. At the beginning of each block, a block cue (word: “Moral” or “Areal”) was shown (7 to 11 s) indicating that this block only comprised a moral or perceptual condition (Fig 1B). We used the word “Areal” instead of “Perceptual” to ensure that both cues contained the same number of letters, thereby improving their visual comparability. The 320 trials were balanced across type of conditions (moral and perceptual) and conflict levels (1, 2, 3, and 4).

To ensure a clear separation between moral and non-moral choices, we used a low-level perceptual task, judging which of two pictures has a larger area, as the non-moral control condition. Although socially relevant control tasks (e.g., judging the gender or race of faces) appear to be appealing alternatives, they risk engaging moral cognition, as prior research has shown that gender and race cues can influence moral judgments [143,144]. Our approach therefore minimizes the risk of unintended conceptual and neural overlap between moral and non-moral processing, enabling a more precise investigation of their distinctions.

If participants switched tasks as required, accuracy should not differ between the first and second halves of trials within task-switching blocks. Consistent with this expectation, we observed no such difference in either the moral condition (*β* = 0.08 ± 0.068, *Z* = 1.20, *P* = 0.232) or the perceptual condition (*β* = −0.13 ± 0.066, *Z* = −1.92, *P* = 0.055), based on hierarchical logistic mixed-effects regressions. These results indicate that participants were able to switch between moral and perceptual choices as required.

### Behavioral analysis

To examine whether participants could accurately estimate the picture sizes, we performed a hierarchical linear mixed-effect regression of the pictures’ actual area sizes (i.e., the percentage of a picture’s pixels that was distinct from the background color, RGB color code = [136, 136, 136]) onto participants’ perceptual ratings (fixed-effect regressor), including participant-specific random constants and random slopes in the model. We performed a z-score normalization on all variables within each participant before the regression analysis.

To test the associations between the picture sizes, perceptual ratings, and moral ratings, we performed two separate hierarchical linear mixed-effect regressions for moral ratings. In one regression, the fixed-effect regressor was perceptual ratings; in the other one, the fixed-effect regressor was pictures’ actual area sizes. In both models, we included participant-specific random constants and random slopes, and we performed a z-score normalization on all variables within each participant before the regression analysis.

For confirming a separation of moral and perceptual choices, we conducted several analyses to test whether each type of choice depended only on the corresponding type of conflict. To examine the influence of different types of conflict on participants’ response time (RT), we performed two separate hierarchical linear mixed-effect regressions for the moral and perceptual conditions, respectively. The fixed-effect regressors included task-relevant conflict (RC, i.e., the moral conflict for the moral choice (RC_moral_) or the perceptual conflict for the perceptual choice (RC_perceptual_)) and task-irrelevant conflict (IC, i.e., the perceptual conflict for the moral choice (IC_moral_) or the moral conflict for the perceptual choice (IC_perceptual_)) (see S5 Fig). Moral conflict was defined as 100% minus the absolute difference in moral ratings between the two pictures within a trial, whereas perceptual conflict was defined analogously as 100% minus the absolute difference in area size between the two pictures within a trial. The fixed effects of the regression models are as follows:


$$\begin{array}{c} Moral\;response\;time\;\sim \;moral\;conflict\;(moral\;task-relevant\;conflict,\;{RC}_{moral})\;\\ +\;perceptual\;conflict\;(moral\;task-irrelevant\;conflict,\;{IC}_{moral}) \end{array}$$

$$\begin{array}{c} Perceptual\;response\;time\;\sim \;moral\;conflict\;(perceptual\;task-irrelevant\;conflict,\;{IC}_{perceptual})\;+\\ perceptual\;conflict\;(perceptual\;task-relevant\;conflict,\;{RC}_{perceptual}) \end{array}$$

To examine the conflict effects on choice accuracy, we conducted two separate hierarchical logistic mixed-effect regressions of choices (1 = correct, 0 = incorrect) for the moral and perceptual conditions. The fixed-effect regressors were the task-relevant conflict and task-irrelevant conflict. We also included response time as a fixed-effect regressor to control for its influence. The fixed effects of the regression models are as follows:


$$\begin{array}{c} Moral\;choice\;accuracy\;\sim \;moral\;conflict\;(moral\;task-relevant\;conflict,\;{RC}_{moral})\;\\ +\;perceptual\;conflict\;(moral\;task-irrelevant\;conflict,\;{IC}_{moral})\;+\;response\;time \end{array}$$

$$\begin{array}{c} Perceptual\;choice\;accuracy\;\sim \;moral\;conflict\;(perceptual\;task-irrelevant\;conflict,\;{IC}_{perceptual})\;\\ +\;perceptual\;conflict\;(perceptual\;task-relevant\;conflict,\;{RC}_{perceptual})\;+\;response\;time \end{array}$$

For all the regressions above, participant-specific random constants and random slopes for each fixed effect were involved as random effects. A z-score normalization was performed on each regressor within each participant before the regression analysis to make the regression parameters comparable.

To further understand the effects of the task-relevant and task-irrelevant conflict, we used Wald chi-squared tests to directly compare their influence (i.e., regression coefficients) on response time and choice accuracy in the moral and perceptual conditions based on the estimates of the mixed-effect regression models above.

### Computational model

To examine the moral and perceptual choices at the algorithmic level, we analyzed participants’ behavioral responses with a prominent type of computational model (the drift-diffusion model, DDM) [64]. This type of model incorporates both choice and response time to decompose the choice process into four main aspects corresponding to different parameters: (1) the efficiency of accumulating evidence (the drift rate, *δ*); (2) the amount of evidence needed to make a choice (the threshold, *α*); (3) the side bias in the choice process (the starting position, *β*); (4) the afferent and efferent delay external to the evidence accumulation process (non-decision time, *τ*). The drift-diffusion model has long been demonstrated performing well in explaining perceptual choice [60,64]. It also reveals its ability to describe moral choice in recent studies [61,63]. Here, we tested four typical versions of the drift-diffusion model for explaining participants’ moral and perceptual behavioral patterns. The models were implemented based on a one-dimensional Wiener process using Just Another Gibbs Sampler (JAGS) [145]:


$$\begin{array}{c} y\;\sim \;Wiener(\;\alpha ,\;\tau ,\;\beta ,\delta ) \end{array}$$

where *y* is the product of empirical choices (*ζ*) and response time. *ζ* was coded as 1 when a choice was correct and as −1 when it was incorrect. As *ζ* was coded based on correctness and no pre-decision evidence existed in our task, we assumed an unbiased diffusion process (i.e., *β* was fixed at the middle point between the boundaries) [146,147].

Each version of the drift-diffusion model has different assumptions about the choice process. Model 1 assumes that the drift rate is constant across trials and the threshold is constant over time (a basic model). Model 2 assumes that the drift rate is modulated by the evidence of different trials [146]:


$$\begin{array}{c} \delta =kE \end{array}$$

where *k* is a parameter that scales the evidence (the drift-rate scale), and *E* represents the evidence, defined as the complement of the task-relevant conflict value. For moral choices, *E* corresponds to the absolute difference in moral ratings between the two pictures; for perceptual choices, it corresponds to the absolute difference in area size between the two pictures. Thus, *E* = 100% − task-relevant conflict = |moral rating_1_ − moral rating_2_| or |area size_1_ − area size_2_| (see S5 Fig). Model 3 assumes that the threshold collapses over time [62]:


$$\begin{array}{c} \alpha ={be}^{-td} \end{array}$$

where *b* is a parameter indicating the initial threshold, *d* is a parameter indicating its exponential rate of decay, and t is the duration of the evidence accumulation (RT − *τ*). Model 4 is a combination of Models 2 and 3, assuming that the drift rate is affected by evidence and the threshold collapses.

We fitted the DDMs separately to each participant’s data in each condition, rather than using a hierarchical Bayesian procedure in which individual-level parameters were jointly estimated as draws from a shared group-level distribution. Specifically, the data were divided by participant and condition, resulting in 106 separate datasets (53 participants × 2 conditions: moral and perceptual). The DDM parameters were then estimated independently for each dataset. This procedure ensured that parameter estimates from different participants and conditions were not constrained by a common hierarchical structure, allowing us to examine individual differences in the correspondence between moral and perceptual DDM parameters across participants. Thus, the observed correlations between moral and perceptual DDM parameters could not be attributed to cross-condition coupling introduced by a joint hierarchical estimation procedure. Posterior inference of the parameters in the Bayesian models was conducted using the Markov Chain Monte Carlo (MCMC) technique. Three chains were run based on different random number generator engines. Each chain started with an initial burn-in step (30,000 iterations) and subsequently generated 30,000 new samples. We applied a thinning of 10 to these samples resulting in a final set of 3,000 samples for each parameter. The thinning step helps to reduce autocorrelation [145]. To check convergence, we inspected the posterior traces and their autocorrelation and conducted Gelman–Rubin tests for each parameter. All parameters had *R̂* < 1.01, suggesting good convergence.

We adopted the leave-one-out cross-validation (LOO), which is suitable for Bayesian model comparison [148], as a measure of goodness-of-fit. The model with the lowest LOO value in the moral or perceptual condition was selected as the winning model. To examine whether the winning model could reproduce key behavioral patterns in the observation, we simulated choices and RT based on the fitted parameters (3,000 simulated samples for each trial) and presented them together with the real data. In both the moral and perceptual conditions, Model 2 won and its simulated data in posterior predictive checks coincided well with the real observation. For further examining the algorithmic similarity, we tested the associations between the winning model’s parameters of moral and perceptual choices using the Pearson correlation.

To assess parameter recoverability, we conducted parameter recovery analyses by refitting the winning model to 20 sets of simulated data for each participant and condition. The simulated data were generated using the posterior mean parameter estimates from the model fitted to the observed data. We evaluated parameter recovery by computing Pearson correlation coefficients between the parameters estimated from the observed data and those recovered from the simulated data. The parameters were highly recoverable in both the moral condition (*r*_drift-rate scale_ = 0.89, 95% CI = [0.88, 0.90]; *r*_threshold_ = 0.83, 95% CI = [0.81, 0.85]; *r*_non-decision time_ = 0.99, 95% CI = [0.99, 0.99]) and the perceptual condition (*r*_drift-rate scale_ = 0.91, 95% CI = [0.90, 0.92]; *r*_threshold_ = 0.91, 95% CI = [0.90, 0.92]; *r*_non-decision time_ = 0.98, 95% CI = [0.98, 0.99]).

### Eye-tracking and pupil dilation

To minimize participants’ eye movements and blinks in the choice task, they were required to keep their eyes on the central letter during the trials and not to blink when the letter was gray. Participants’ fixation patterns and pupil sizes during the choice task (but not the rating task) were recorded by an MR-compatible Eyelink 1,000 eye-tracker system (https://www.sr-research.com/). As these data are not directly related to the current study, we do not report them here.

### MRI data acquisition

Whole-brain imaging data were acquired at the Laboratory for Social and Neural Systems Research of the Zurich Center for Neuroeconomics, Department of Economics, University of Zurich using a Philips Achieva 3-Tesla whole-body magnetic resonance scanner equipped with a 32-channel head coil. During the choice task, T2-weighted echo-planar functional images were collected (TR/TE: 2334/30 ms; FoV: [240 mm (RL) 240 mm (AP) 140 mm (FH)]; flip angle: 90°; voxel size: 3 × 3 × 3 mm^3^; number of slices: 40; slice gap: 0.5 mm; slice scan order: ascending; number of sessions: 10; duration of a session: 5.6 min). Additionally, a T1-weighted 3-dimensional fast-field echo structural image was collected for image registration (TR/TE: 8.3/3.9 ms; FoV: [181 mm (RL) 256 mm (AP) 256 mm (FH)]; flip angle: 8°; voxel size: 1 × 1 × 1 mm^3^; number of slices: 181) and multi-slice gradient-echo B0 images (field maps) were acquired for correcting deformations (TR/TE1/TE2: 1150/4.6/6.9 ms; FoV: [240 mm (RL) 240 mm (AP) 150 mm (FH)]; flip angle: 72°; voxel size: 3 × 3 × 3 mm^3^; number of slices: 50; slice gap: 0 mm; slice scan order: ascending).

### fMRI data preprocessing

Imaging data were preprocessed by Statistical Parametric Mapping 12 (SPM 12, https://www.fil.ion.ucl.ac.uk/spm/software/spm12/). Functional images were realigned to the first volume for head-motion correction, unwrapped for geometric distortions correction using field maps, slice-time corrected to the acquisition time of the middle slice. The mean functional image (as source image) was co-registered with each participant’s high-resolution T1-weighted image (as reference image) using parameters from the segmentation performed on both types of images. The functional images were then normalized into the Montreal Neurological Institute (MNI) space, resampled to 3-mm isotropic voxels, detrended, and smoothed using an 8-mm full width at half maximum (FWHM) Gaussian kernel.

### Univariate activation analysis

Using SPM 12, we built a general linear model (GLM 1) to identify regions responding parametrically to task-relevant conflict values, task-irrelevant conflict values, and response time in the moral and perceptual conditions, respectively (i.e., the main analysis). The GLM contained two main event regressors describing the onsets of (1) moral choices and (2) perceptual choices (the presence of the two pictures; duration: 0 s). Each of them was associated with three parametric modulators: task-relevant conflict (RC), task-irrelevant conflict (IC), and response time (RT). The parametric modulators were not orthogonalized, thereby ensuring that they could capture unique variance. We involved task-irrelevant conflict and response time as parametric modulators for controlling and investigating the effects of no interest (i.e., the effect of IC) and domain-general basic psychophysiological processes (indicated by RT). In line with the behavioral analysis, z-score normalization was performed on each variable. The GLM contained five additional regressors of no interest, indicating the onsets of moral or perceptual block cues (the gray words, duration: 7–11 s), moral or perceptual trial cues (the gray letter, duration: 1 s), missing trials (if existed). These regressors were convolved with a canonical hemodynamic response function. Six head motion parameters from the realignment procedure were included in the GLM as nuisance regressors controlling for a motion effect of no interest. Nine contrasts were performed at the first level, including parametric contrasts for each modulator in each condition (six contrasts), the difference between task-relevant-conflict effects in the moral and perceptual conditions (RC_moral_ versus RC_perceptual_), the difference between task-irrelevant-conflict effects in the moral and perceptual conditions (IC_moral_ versus IC_perceptual_), and the difference between response-time effects in the moral and perceptual conditions (RT_moral_ versus RT_perceptual_). The contrast images from the first-level analysis were entered into second-level one-sample *T* tests.

To identify common regions responding parametrically to task-relevant conflict, task-irrelevant conflict, and response time in both moral and perceptual conditions, we performed three conjunction analyses, including RC_moral_ ∩ RC_perceptual_, IC_moral_ ∩ IC_perceptual_, and RT_moral_ ∩ RT_perceptual_ [149].

As a control analysis, we built another three GLMs to examine to what extent involving RC, IC, and RT as parametric modulators in the same GLM influenced the neural findings compared to putting them in different GLMs. The difference between the new GLMs and GLM 1 was that each of them had one parametric modulator (GLM 2: RC, GLM 3: IC, GLM 4: RT) instead of three (GLM 1: RC, IC, and RT). The findings based on GLMs 2, 3, and 4 (S9 and S10 Tables) were similar to those based on GLM 1 (S5 and S8 Tables).

In another control analysis, we only analyzed the trials in which participants made a correct choice using GLM 1. It helps to control the potential effect of error on our neural results. The findings of this control analysis (S11 and S12 Tables) were similar to those of the main analysis (S5 and S8 Tables).

In addition, to examine potential nonlinear relationships between task-relevant conflict and brain activation, we constructed an additional model (GLM 5). This model extended GLM 1 by including the quadratic and cubic terms of RC as parametric modulators and by applying orthogonalization to the modulators [150]. The modulators were entered into the GLM in the following order: IC, RT, RC, quadratic RC, and cubic RC.

To identify brain activation related to individual differences in drift-diffusion model parameters, we performed an additional second-level analysis. We conducted one-sample *T* tests at the second level using the RC_moral_- and RC_perceptual_-related first-level parametric contrast images from GLM 1, including individual moral and perceptual drift-rate scales and thresholds as covariates. At the second level, we tested whether these conflict-related parametric effects were associated with each DDM parameter. Moreover, to determine whether brain activation associated with perceptual drift-diffusion parameters differed from that associated with moral drift-diffusion parameters, we explicitly defined contrasts at the second level comparing corresponding parameters (i.e., moral drift-rate scale versus perceptual drift-rate scale and moral threshold versus perceptual threshold).

To identify brain regions responding parametrically to chosen value, we constructed an additional general linear model (GLM 6) based on GLM 1. GLM 6 differed from GLM 1 only in the parametric modulators. Specifically, instead of the three parametric modulators used in GLM 1, GLM 6 included two parametric modulators for each main event regressor: chosen value (CV) and response time. In the moral condition, chosen value was defined as the moral rating of the selected picture (CV_moral_). In the perceptual condition, chosen value was defined as the area size of the selected picture (CV_perceptual_). At the first level, we constructed contrasts for the parametric effects of CV_moral_ and CV_perceptual_ on BOLD activity, as well as the difference between these effects (CV_moral_ versus CV_perceptual_). The contrast images from the first-level analysis were entered into second-level one-sample *T* tests. We also conducted a conjunction analysis to identify regions commonly associated with CV_moral_ and CV_perceptual_ [149].

We set the statistical threshold to *P* < 0.05 (FWE-corrected at the cluster level) with an initial cluster-defining voxel-level threshold of *P* < 0.001 for the whole-brain analysis.

### Region-of-interest analyses

#### Correlations between the neural activation associated with the moral task-relevant conflict and perceptual task-relevant conflict.

To explore potential associations between the neural activation related to the moral and perceptual task-relevant conflict at the individual level, we defined seven clusters that had significant responses to the moral or perceptual task-relevant conflict as the regions of interest (ROIs) (i.e., rpTPJ, DLPFC, DMPFC, raTPJ, lTPJ, and bilateral OC) (Fig 3A and 3B). We extracted the beta estimates from the RC_moral_-related parametric contrast images for the rpTPJ, DLPFC, and DMPFC clusters and from the RC_perceptual_-related parametric contrast images for the raTPJ, lTPJ, and bilateral OC clusters. We calculated the Pearson correlations among the beta estimates (S6 Table). The false discovery rate correction was adopted for controlling the type I error (*q*-value: 0.05).

#### Neural implementation of moral and perceptual conflict in the brain areas related to morality.

To further confirm that morality-related brain regions proposed by previous studies have different representations of moral and perceptual conflict, we conducted an ROI analysis. The ROIs were constructed as spheres with 8-mm radius centered at the peak voxels of clusters reported in a recent meta-analysis on morality [70]. The ROIs included right TPJ (MNI coordinates: [54, −56, 26]), left TPJ ([−48, −60, 24]), MPFC ([2, 60, 14]), orbitofrontal cortex ([−36, 20, −10]), precuneus ([2, −58, 34]), and amygdala ([−22, −6, −16]). We extracted the contrast values from the RC_moral_- and RC_perceptual_-related parametric contrast images for each ROI, and compared them with each other using a paired-samples *T* test. The false discovery rate correction was adopted for controlling the type I error (*q*-value: 0.05).

#### Gradient effect.

For a more quantitative examination of the spatial segregation within the TPJ, we defined three ROIs along the right posterior-to-anterior TPJ (Fig 4C). The ROIs were created as 3-mm spheres centered on the peaks of the rpTPJ and raTPJ clusters identified by the RC_moral_- and RC_perceptual_-related parametric contrasts in the univariate activation analysis and the midpoint between the two peaks. The MNI coordinates of the three ROIs were: [42, −60, 42], [51, −54, 36], [60, −48, 33]. The location of the midpoint was slightly adjusted, as the functional images were resampled to 3-mm isotropic voxels. The mathematical midpoint (i.e., [51, −54, 37.5]) is thus meaningless. To examine how these ROIs respond to moral and perceptual task-relevant conflict, we extracted the beta estimates from the RC_moral_- and RC_perceptual_-related parametric contrast images for each ROI and visualized them. No further analysis was conducted on these beta estimates to avoid circular analysis [75].

All the ROI analyses were conducted based on the results of GLM 1.

### Functional connectivity analysis

The results of univariate activation analysis showed that brain regions representing the moral and perceptual task-relevant conflict were different. To extend our analysis to brain circuits and identify functional connectivity related to task-relevant conflict, we conducted psychophysiological interaction (PPI) analysis using an SPM-based PPI toolbox (http://www.nitrc.org/projects/gppi, version 13.1) [151]. We used the seven clusters that responded parametrically to the moral task-relevant conflict (rpTPJ, DLPFC, and DMPFC) or perceptual task-relevant conflict (raTPJ, lTPJ, and bilateral OC) as seed regions (see Fig 3A and 3B). The physiological factors were constructed by extracting and deconvolving participants’ first eigenvariate of the functional time course from the seed regions. The psychological factors were defined in the same way as GLM 1 of the univariate activation analysis. Thus, task-relevant conflict, task-irrelevant conflict, and response time were all included in the PPI GLM as parametric modulators. The psychophysiological factors resulted from convolving the product of the psychological and physiological factors (RC_moral_×eigenvariate and RC_perceptual_×eigenvariate) with a canonical hemodynamic response. We added all factors to the first-level design matrix for parameter estimation. Six estimated head movement parameters were included to regress out any head-motion artifacts. For the PPI analysis, we constructed three first-level contrasts testing moral-conflict-dependent connectivity, perceptual-conflict-dependent connectivity, and the difference between these effects (RC_moral_ versus RC_perceptual_). The contrast images from the first-level analysis were entered into one-sample *T* tests at the second level.

Additionally, to identify functional connectivity related to individual differences in drift-diffusion model parameters, we performed an additional second-level analysis. We conducted one-sample *T* tests at the second level using the moral- and perceptual-conflict-dependent first-level PPI contrast images described above, including individual moral and perceptual drift-rate scales and thresholds as covariates. At the second level, we tested whether conflict-dependent connectivity effects were associated with each DDM parameter. Moreover, to determine whether functional connectivity associated with moral drift-diffusion parameters differs from that associated with perceptual drift-diffusion parameters, we explicitly defined contrasts at the second level comparing corresponding parameters (i.e., moral drift-rate scale versus perceptual drift-rate scale and moral threshold versus perceptual threshold).

PPI results were thresholded using voxel-level *P* < 0.001 (uncorrected) combined with cluster-level *P* < 0.05 (FWE-corrected) for the whole-brain analysis.

## Supporting information

S1 Fig Examples of the moral (A) and perceptual (B) ratings. Participants rated the same set of pictures according to different questions. They could change the place of the indicator (the round dot) by moving a computer mouse and confirm their decision by pressing the left button of the mouse.
(TIF)

S2 Fig Each participant’s perceptual ratings and actual area sizes (i.e., the percentage of a picture’s pixels that was distinct from the background color, RGB color code = [136, 136, 136]) are shown. The parameter estimates of a hierarchical linear mixed-effect regression revealed that the perceptual ratings and actual area sizes were highly correlated (fixed effect: *β* = 0.86 ± 0.009, *P* < 0.0001), suggesting that participants could make accurate perceptual estimations. Each red line represents the least squares fit of a participant (for visual display).
(TIF)

S3 Fig Each participant’s perceptual ratings and moral ratings are shown. The parameter estimates of a hierarchical linear mixed-effect regression revealed that they were not significantly correlated (fixed effect: *β* = 0.02 ± 0.016, *P* = 0.197). Each red line represents the least squares fit of a participant (for visual display).
(TIF)

S4 Fig The actual area sizes and moral ratings are shown. The parameter estimates of a hierarchical linear mixed-effect regression revealed that they were not significantly correlated (fixed effect: *β* = −0.01 ± 0.016, *P* = 0.498). Each red line represents the least squares fit of a participant (for visual display).
(TIF)

S5 Fig An illustration of the relationships among the moral rating, area size, moral conflict, perceptual conflict, task-relevant conflict, task-irrelevant conflict, and evidence. It is noted that all conflict values were z-normalized for regression analyses.
(TIF)

S6 Fig The neural representation of the moral and perceptual task-relevant conflict in the brain areas related to morality. A meta-analysis has associated the activation of the right temporoparietal junction (rTPJ), left temporoparietal junction (lTPJ), medial prefrontal cortex (MPFC), orbitofrontal cortex (OFC), precuneus, and amygdala with morality [70]. We defined these regions as the regions of interest (ROIs). In line with the whole-brain analysis, we found that the bilateral TPJ had stronger positive parametric responses to the task-relevant conflict in the moral condition (RC_moral_) compared to that in the perceptual condition (RC_perceptual_). The locations of the ROIs are displayed. The mean beta estimates of ROIs in the moral and perceptual conditions are shown. Error bars represent the standard errors. Each dot indicates the estimate of an individual participant. ^*^P_FDR corrected_ < 0.05; ^***^P_FDR corrected_ < 0.001. The underlying data for this figure are available in the “Data” folder of our OSF repository.
(TIF)

S7 Fig Results of main and control analyses to establish neural specificity of the moral and perceptual task-relevant conflict processing. (**A**) Results of the main analysis based on GLM 1 and all trials. (**B**) Results of the main analysis based on GLM 2 and all trials. (**C**) Results of the main analysis based on GLM 1 and correct trials. RC_moral_, moral task-relevant conflict; RC_perceptual_, perceptual task-relevant conflict; TPJ, temporoparietal junction; DLPFC, dorsolateral prefrontal cortex; VLPFC, ventrolateral prefrontal cortex; OC, occipital cortex; ITC, inferior temporal cortex; r, right; l, left; a, anterior; p, posterior. Whole-brain FWE-cluster correction at *P* < 0.05 after cluster-forming threshold at *P* < 0.001.
(TIF)

S8 Fig Illustration of how the parametric modulation analysis reduces the potential confounding effect of abstraction level. (**A**) A simple contrast between the moral and perceptual conditions would compare trials that differ not only in task domain but also in overall abstraction level. Such a comparison would therefore be more vulnerable to influence by abstraction level. (**B**) In our analyses, task-relevant conflict was modeled parametrically within each condition. Thus, higher moral-conflict trials were effectively compared with lower moral-conflict trials within the moral condition, and higher perceptual-conflict trials were effectively compared with lower perceptual-conflict trials within the perceptual condition. Because abstraction level is likely to be more comparable within each task domain than between moral and perceptual tasks as a whole, this approach reduces the likelihood that the observed conflict-related neural effects are driven solely by overall differences in abstraction level. RC_moral_, task-relevant conflict in the moral condition; RC_perceptual_, task-relevant conflict in the perceptual condition.
(TIF)

S1 Table Linear mixed-effect regression of response time in the moral condition. (DOCX)

S2 Table Linear mixed-effect regression of response time in the perceptual condition. (DOCX)

S3 Table Logistic mixed-effect regression of choice accuracy in the moral condition. (DOCX)

S4 Table Logistic mixed-effect regression of choice accuracy in the perceptual condition. (DOCX)

S5 Table Brain regions responding parametrically to the moral or perceptual task-relevant conflict and having differential sensitivity to the moral and perceptual task-relevant conflict when task-relevant conflict, task-irrelevant conflict, and response time were all included in a general linear model (results based on GLM 1 and all trials). (DOCX)

S6 Table Pearson correlations between the neural activation associated with the moral and perceptual task-relevant conflict. (DOCX)

S7 Table Statistical results of the paired-samples *T* tests between neural responses to the moral and perceptual task-relevant conflict in the brain regions related to morality. (DOCX)

S8 Table Brain regions responding parametrically to the moral and/or perceptual response time and having differential sensitivity to the moral and perceptual response time when task-relevant conflict, task-irrelevant conflict, and response time were all included in a general linear model (results based on GLM 1 and all trials). (DOCX)

S9 Table Brain regions responding parametrically to the moral or perceptual task-relevant conflict and having differential sensitivity to the moral and perceptual task-relevant conflict when task-relevant conflict was in an independent general linear model (results based on GLM 2 and all trials). (DOCX)

S10 Table Brain regions responding parametrically to the moral and/or perceptual response time and having differential sensitivity to the moral and perceptual response time when response time was in an independent general linear model (results based on GLM 4 and all trials). (DOCX)

S11 Table Brain regions responding parametrically to the moral or perceptual task-relevant conflict and having differential sensitivity to the moral and perceptual task-relevant conflict when task-relevant conflict, task-irrelevant conflict, and response time were all included in a general linear model (results based on GLM 1 and correct trials). (DOCX)

S12 Table Brain regions responding parametrically to the moral and/or perceptual response time and having differential sensitivity to the moral and perceptual response time when task-relevant conflict, task-irrelevant conflict, and response time were all included in a general linear model (results based on GLM 1 and correct trials). (DOCX)

S13 Table Brain regions showing parametric associations with moral and/or perceptual chosen value and their domain-specific sensitivity. (DOCX)

S14 Table Functional connectivity responding parametrically to the moral task-relevant conflict and having differential sensitivity to the moral and perceptual task-relevant conflict. (DOCX)

S15 Table Brain activation associated with perceptual threshold. (DOCX)

S16 Table Functional connectivity associated with different drift-diffusion model parameters. (DOCX)

S17 Table Descriptive results of the picture ratings. (DOCX)

## Abbreviations

CV: chosen value
DDM: drift diffusion model
fMRI: functional magnetic resonance imaging
FWHM: full width at half maximum
GLM: general linear model
IC: irrelevant conflict
ITC: inferior temporal cortex
JAGS: Just Another Gibbs Sampler
lOC: left occipital cortex
LOO: leave-one-out cross-validation
LPFC: lateral prefrontal cortex
lTPJ: left temporoparietal junction
MCC: middle cingulate cortex
MCMC: Markov Chain Monte Carlo
MNI: Montreal Neurological Institute
MPFC: medial prefrontal cortex
MVPA: multivariate pattern analysis
OC: occipital cortex
OFC: orbitofrontal cortex
PCC: posterior cingulate cortex
PPI: psychophysiological interaction
raTPJ: right anterior temporoparietal junction
RC: relevant conflict
rOC: right occipital cortex
ROI: region of interest
rpTPJ: right posterior temporoparietal junction
RT: response time
rTPJ: right temporoparietal junction
SPC: superior parietal cortex
tDCS: transcranial direct current stimulation
TMS: transcranial magnetic stimulation
TPJ: temporoparietal junction
