# Supplementary material for: Moral and perceptual conflict processing are implemented by distinct brain activation and functional connectivity patterns
Source: PLoS Biol. 2026 Oct 5;24(10):e3004025. doi: 10.1371/journal.pbio.3004025 (PMC13637876; doi:10.1371/journal.pbio.3004025)
Supplement: Supplementary file 1 [file pbio.3004025.s009.docx]

**S1 Table.** Linear mixed-effect regression of response time in the moral condition.

| Fixed-effect regressor | Estimate | Standard error | *T* | *P* |
| --- | --- | --- | --- | --- |
| Moral conflict | 0.037 | 0.006 | 6.11 | < 0.001 |
| Perceptual conflict | 0.007 | 0.005 | 1.49 | 0.135 |
| (Intercept) | 1.532 | 0.032 | 48.49 | < 0.001 |
