# Supplementary material for: Moral and perceptual conflict processing are implemented by distinct brain activation and functional connectivity patterns
Source: PLoS Biol. 2026 Oct 5;24(10):e3004025. doi: 10.1371/journal.pbio.3004025 (PMC13637876; doi:10.1371/journal.pbio.3004025)
Supplement: Supplementary file 2 [file pbio.3004025.s010.docx]

**S2 Table.** Linear mixed-effect regression of response time in the perceptual condition.

| Fixed-effect regressor | Estimate | Standard error | *T* | *P* |
| --- | --- | --- | --- | --- |
| Moral conflict | 0.003 | 0.004 | 0.80 | 0.427 |
| Perceptual conflict | 0.106 | 0.007 | 14.56 | < 0.001 |
| (Intercept) | 1.273 | 0.034 | 37.62 | < 0.001 |
