# Supplementary material for: Moral and perceptual conflict processing are implemented by distinct brain activation and functional connectivity patterns
Source: PLoS Biol. 2026 Oct 5;24(10):e3004025. doi: 10.1371/journal.pbio.3004025 (PMC13637876; doi:10.1371/journal.pbio.3004025)
Supplement: Supplementary file 3 [file pbio.3004025.s011.docx]

**S3 Table.** Logistic mixed-effect regression of choice accuracy in the moral condition.

| Fixed-effect regressor | Estimate | Standard error | *Z* | *P* |
| --- | --- | --- | --- | --- |
| Moral conflict | -0.37 | 0.03 | -13.76 | < 0.001 |
| Perceptual conflict | -0.04 | 0.03 | -1.49 | 0.136 |
| Response time | -0.26 | 0.03 | -7.72 | < 0.001 |
| (Intercept) | 0.97 | 0.03 | 38.05 | < 0.001 |
