# Supplementary material for: Moral and perceptual conflict processing are implemented by distinct brain activation and functional connectivity patterns
Source: PLoS Biol. 2026 Oct 5;24(10):e3004025. doi: 10.1371/journal.pbio.3004025 (PMC13637876; doi:10.1371/journal.pbio.3004025)
Supplement: Supplementary file 4 [file pbio.3004025.s012.docx]

**S4 Table.** Logistic mixed-effect regression of choice accuracy in the perceptual condition.

| Fixed-effect regressor | Estimate | Standard error | *Z* | *P* |
| --- | --- | --- | --- | --- |
| Moral conflict | 0.01 | 0.03 | 0.36 | 0.72 |
| Perceptual conflict | -1.00 | 0.06 | -16.21 | < 0.001 |
| Response time | -0.33 | 0.03 | -9.89 | < 0.001 |
| (Intercept) | 1.71 | 0.06 | 27.02 | < 0.001 |
