# Supplementary material for: Moral and perceptual conflict processing are implemented by distinct brain activation and functional connectivity patterns
Source: PLoS Biol. 2026 Oct 5;24(10):e3004025. doi: 10.1371/journal.pbio.3004025 (PMC13637876; doi:10.1371/journal.pbio.3004025)
Supplement: Supplementary file 5 [file pbio.3004025.s013.docx]

**S5 Table.** Brain regions responding parametrically to the moral or perceptual task-relevant conflict and having differential sensitivity to the moral and perceptual task-relevant conflict when task-relevant conflict, task-irrelevant conflict, and response time were all included in a general linear model (results based on GLM 1 and all trials).

| Contrast/Region | Cluster Size | Peak MNI Coordinates | | | *T* | *P*_FWE_ |
| --- | --- | --- | --- | --- | --- | --- |
|  |  | x | y | z |  |  |
| ***RC_moral_*** |  |  |  |  |  |  |
| rpTPJ | 135 | 42 | -60 | 42 | 4.86 | 0.019 |
| DLPFC | 130 | 51 | 30 | 24 | 4.40 | 0.022 |
| DMPFC | 246 | -3 | 24 | 45 | 4.57 | 0.001 |
| ***RC_perceptual_*** |  |  |  |  |  |  |
| rOC (extending to ITC and SPC) | 930 | 45 | -66 | -6 | 5.65 | <0.001 |
| lOC (extending to ITC and SPC) | 643 | -39 | -87 | 9 | 6.23 | <0.001 |
| ***-RC_perceptual_*** |  |  |  |  |  |  |
| raTPJ | 192 | 60 | -48 | 33 | 5.35 | 0.005 |
| lTPJ | 385 | -57 | -48 | 39 | 7.31 | <0.001 |
| ***RC_moral_ > RC_perceptual_*** |  |  |  |  |  |  |
| rTPJ | 334 | 51 | -51 | 30 | 6.14 | <0.001 |
| lTPJ | 401 | -57 | -48 | 36 | 5.80 | <0.001 |
| DLPFC | 170 | 39 | 12 | 42 | 4.80 | 0.008 |
| VLPFC | 103 | -45 | 24 | 3 | 4.33 | 0.048 |
| ***RC_perceptual_ > RC_moral_*** |  |  |  |  |  |  |
| rOC (extending to SPC) | 368 | 27 | -72 | 30 | 5.60 | <0.001 |
| ITC | 171 | 48 | -60 | -9 | 5.25 | 0.008 |
| lOC (extending to ITC) | 523 | -39 | -87 | 9 | 5.40 | <0.001 |

Note: RC_moral_, moral task-relevant conflict; RC_perceptual_, perceptual task-relevant conflict; Contrast labels without a minus sign indicate positive parametric associations with BOLD activity; labels with a minus sign indicate negative parametric associations. TPJ, temporoparietal junction; DLPFC, dorsolateral prefrontal cortex; DMPFC, dorsomedial prefrontal cortex; OC, occipital cortex; ITC, inferior temporal cortex; SPC, superior parietal cortex; VLPFC, ventrolateral prefrontal cortex; r, right; l, left; a, anterior; p, posterior; MNI, Montreal Neurological Institute; FWE, family-wise error correction.
