# Supplementary material for: Moral and perceptual conflict processing are implemented by distinct brain activation and functional connectivity patterns
Source: PLoS Biol. 2026 Oct 5;24(10):e3004025. doi: 10.1371/journal.pbio.3004025 (PMC13637876; doi:10.1371/journal.pbio.3004025)
Supplement: Supplementary file 6 [file pbio.3004025.s014.docx]

**S6 Table.** Pearson correlations between the neural activation associated with the moral and perceptual task-relevant conflict.

| Regions responding to moral task-relevant conflict | Regions responding to perceptual task-relevant conflict | *R* | *P* | *P*_FDR_ |
| --- | --- | --- | --- | --- |
| rpTPJ | raTPJ | 0.138 | 0.325 | 0.650 |
| rpTPJ | lTPJ | 0.019 | 0.891 | 0.933 |
| rpTPJ | rOC | -0.063 | 0.653 | 0.871 |
| rpTPJ | lOC | -0.012 | 0.933 | 0.933 |
| DLPFC | raTPJ | 0.105 | 0.454 | 0.681 |
| DLPFC | lTPJ | -0.111 | 0.428 | 0.681 |
| DLPFC | rOC | -0.162 | 0.246 | 0.590 |
| DLPFC | lOC | -0.205 | 0.140 | 0.420 |
| DMPFC | raTPJ | 0.261 | 0.059 | 0.354 |
| DMPFC | lTPJ | 0.024 | 0.862 | 0.933 |
| DMPFC | rOC | -0.283 | 0.040 | 0.354 |
| DMPFC | lOC | -0.211 | 0.130 | 0.420 |

Note: TPJ, temporoparietal junction; DLPFC, dorsolateral prefrontal cortex; DMPFC, dorsomedial prefrontal cortex; OC, occipital cortex; r, right; l, left; a, anterior; p, posterior. FDR, false discovery rate correction (q-value: 0.05).
