# Supplementary material for: Moral and perceptual conflict processing are implemented by distinct brain activation and functional connectivity patterns
Source: PLoS Biol. 2026 Oct 5;24(10):e3004025. doi: 10.1371/journal.pbio.3004025 (PMC13637876; doi:10.1371/journal.pbio.3004025)
Supplement: Supplementary file 7 [file pbio.3004025.s015.docx]

**S7 Table.** Statistical results of the paired-samples *T* tests between neural responses to the moral and perceptual task-relevant conflict in the brain regions related to morality.

| Regions related to morality | *T* | *P* | *P*_FDR_ |
| --- | --- | --- | --- |
| rTPJ | 4.98 | <0.001 | <0.001 |
| lTPJ | 2.93 | 0.005 | 0.015 |
| MPFC | 1.64 | 0.106 | 0.159 |
| OFC | 0.50 | 0.622 | 0.622 |
| Precuneus | 0.86 | 0.395 | 0.474 |
| Amygdala | -1.90 | 0.063 | 0.125 |

Note: TPJ, temporoparietal junction; MPFC, medial prefrontal cortex; OFC, orbitofrontal cortex; r, right; l, left; FDR, false discovery rate correction (q-value: 0.05).
