# Supplementary material for: Moral and perceptual conflict processing are implemented by distinct brain activation and functional connectivity patterns
Source: PLoS Biol. 2026 Oct 5;24(10):e3004025. doi: 10.1371/journal.pbio.3004025 (PMC13637876; doi:10.1371/journal.pbio.3004025)
Supplement: Supplementary file 8 [file pbio.3004025.s016.docx]

**S8 Table.** Brain regions responding parametrically to the moral and/or perceptual response time and having differential sensitivity to the moral and perceptual response time when task-relevant conflict, task-irrelevant conflict, and response time were all included in a general linear model (results based on GLM 1 and all trials).

| Contrast/Region | Cluster Size | Peak MNI Coordinates | | | *T* | *P*_FWE_ |
| --- | --- | --- | --- | --- | --- | --- |
|  |  | x | y | z |  |  |
| ***RT_moral_*** |  |  |  |  |  |  |
| MCC (extending to insula and LPFC) | 2764 | -3 | 12 | 51 | 11.77 | <0.001 |
| OC (extending to precuneus) | 1545 | -39 | -81 | 27 | 7.18 | <0.001 |
| Insula (extending to LPFC) | 824 | -30 | 24 | 3 | 9.99 | <0.001 |
| Caudate | 106 | 12 | 9 | 6 | 6.96 | 0.044 |
| ***RT_perceptual_*** |  |  |  |  |  |  |
| MCC | 543 | -6 | 9 | 48 | 9.69 | <0.001 |
| Insula (extending to LPFC) | 612 | 30 | 24 | 6 | 7.54 | <0.001 |
| Insula (extending to LPFC, POG, precuneus, OC) | 2986 | -30 | 27 | 3 | 9.06 | <0.001 |
| POG | 173 | 42 | -33 | 42 | 5.38 | 0.007 |
| ITC | 242 | -51 | -57 | 0 | 6.30 | 0.002 |
| Thalamus | 243 | -9 | -15 | -6 | 5.71 | 0.002 |
| ***RT_perceptual_ > RT_moral_*** |  |  |  |  |  |  |
| POG | 350 | -54 | -33 | 45 | 7.19 | <0.001 |
| ***RT_moral_ ∩ RT_perceptual_*** |  |  |  |  |  |  |
| MCC | 616 | -6 | 9 | 51 | 9.01 | <0.001 |
| OC (extending to precuneus) | 438 | -27 | -75 | 39 | 5.45 | <0.001 |
| Insula (extending to LPFC) | 618 | 33 | 24 | 3 | 6.88 | <0.001 |
| Insula (extending to LPFC) | 724 | -30 | 24 | 3 | 9.21 | <0.001 |
| LPFC | 377 | -30 | 3 | 60 | 6.12 | <0.001 |

Note: RT_moral_, moral response time; RT_perceptual_, perceptual response time; Contrast labels indicate positive parametric associations with BOLD activity. MCC, middle cingulate cortex; LPFC, lateral prefrontal cortex; OC, occipital cortex; POG, postcentral gyrus; ITC, inferior temporal cortex; MNI, Montreal Neurological Institute; FWE, family-wise error correction.
