# Supplementary material for: Moral and perceptual conflict processing are implemented by distinct brain activation and functional connectivity patterns
Source: PLoS Biol. 2026 Oct 5;24(10):e3004025. doi: 10.1371/journal.pbio.3004025 (PMC13637876; doi:10.1371/journal.pbio.3004025)
Supplement: Supplementary file 9 [file pbio.3004025.s017.docx]

**S9 Table.** Brain regions responding parametrically to the moral or perceptual task-relevant conflict and having differential sensitivity to the moral and perceptual task-relevant conflict when task-relevant conflict was in an independent general linear model (results based on GLM 2 and all trials).

| Contrast/Region | Cluster Size | Peak MNI Coordinates | | | *T* | *P*_FWE_ |
| --- | --- | --- | --- | --- | --- | --- |
|  |  | x | y | z |  |  |
| ***RC_moral_*** |  |  |  |  |  |  |
| rpTPJ | 141 | 42 | -60 | 39 | 4.87 | 0.015 |
| DMPFC (extending to DLPFC) | 386 | -3 | 24 | 42 | 5.00 | <0.001 |
| VLPFC | 255 | -42 | 18 | 24 | 4.61 | 0.001 |
| ***RC_perceptual_*** |  |  |  |  |  |  |
| rOC (extending to ITC and SPC) | 1625 | 27 | -72 | 42 | 7.12 | <0.001 |
| lOC (extending to ITC and SPC) | 1333 | -21 | -69 | 48 | 7.15 | <0.001 |
| DMPFC (extending to ACC) | 272 | -3 | 21 | 42 | 7.24 | 0.001 |
| PRG | 153 | 51 | 9 | 30 | 4.98 | 0.012 |
| ***-RC_perceptual_*** |  |  |  |  |  |  |
| raTPJ | 708 | 60 | -45 | 36 | 6.07 | <0.001 |
| lTPJ | 595 | -57 | -48 | 36 | 7.76 | <0.001 |
| MCC | 164 | 12 | -42 | 36 | 4.27 | 0.009 |
| ACC | 185 | -15 | 45 | 0 | 4.41 | 0.005 |
| ***RC_moral_ > RC_perceptual_*** |  |  |  |  |  |  |
| rTPJ | 472 | 51 | -51 | 30 | 7.78 | <0.001 |
| lTPJ | 500 | -45 | -57 | 39 | 6.52 | <0.001 |
| DLPFC | 183 | 42 | 15 | 45 | 4.98 | 0.006 |
| ***RC_perceptual_ > RC_moral_*** |  |  |  |  |  |  |
| rOC (extending to SPC) | 744 | 24 | -75 | 48 | 6.07 | <0.001 |
| ITC | 218 | 51 | -57 | -9 | 5.66 | 0.003 |
| lOC (extending to ITC and SPC) | 953 | -27 | -72 | 27 | 7.36 | <0.001 |

Note: RC_moral_, moral task-relevant conflict; RC_perceptual_, perceptual task-relevant conflict; Contrast labels without a minus sign indicate positive parametric associations with BOLD activity; labels with a minus sign indicate negative parametric associations. TPJ, temporoparietal junction; DLPFC, dorsolateral prefrontal cortex; DMPFC, dorsomedial prefrontal cortex; VLPFC, ventrolateral prefrontal cortex; OC, occipital cortex; ITC, inferior temporal cortex; ACC, anterior cingulate cortex; PRG, precentral gyrus; MCC, middle cingulate cortex; SPC, superior parietal cortex; r, right; l, left; a, anterior; p, posterior; MNI, Montreal Neurological Institute; FWE, family-wise error correction.
