# Supplementary material for: Moral and perceptual conflict processing are implemented by distinct brain activation and functional connectivity patterns
Source: PLoS Biol. 2026 Oct 5;24(10):e3004025. doi: 10.1371/journal.pbio.3004025 (PMC13637876; doi:10.1371/journal.pbio.3004025)
Supplement: Supplementary file 10 [file pbio.3004025.s018.docx]

**S10 Table.** Brain regions responding parametrically to the moral and/or perceptual response time and having differential sensitivity to the moral and perceptual response time when response time was in an independent general linear model (results based on GLM 4 and all trials).

| Contrast/Region | Cluster Size | Peak MNI Coordinates | | | *T* | *P*_FWE_ |
| --- | --- | --- | --- | --- | --- | --- |
|  |  | x | y | z |  |  |
| ***RT_moral_*** |  |  |  |  |  |  |
| MCC (extending to insula and LPFC) | 3819 | -3 | 12 | 51 | 13.21 | <0.001 |
| OC (extending to precuneus) | 1124 | 9 | -54 | 15 | 7.68 | <0.001 |
| OC | 529 | -36 | -84 | 27 | 8.27 | <0.001 |
| Caudate | 240 | 12 | 9 | 3 | 7.62 | 0.002 |
| ***RT_perceptual_*** |  |  |  |  |  |  |
| MCC (extending to insula, LPFC, POG, precuneus, OC) | 4471 | -6 | 9 | 51 | 11.03 | <0.001 |
| Insula (extending to LPFC) | 751 | 30 | 24 | 3 | 8.34 | <0.001 |
| LPFC | 150 | 27 | 0 | 57 | 5.36 | 0.015 |
| POG | 294 | 45 | -33 | 45 | 6.79 | 0.001 |
| Precuneus | 584 | 21 | -66 | 30 | 5.79 | <0.001 |
| ITC | 192 | 42 | -51 | -12 | 5.36 | 0.005 |
| Thalamus (extending to brainstem) | 418 | 6 | -27 | -6 | 6.22 | <0.001 |
| ***RT_perceptual_ > RT_moral_*** |  |  |  |  |  |  |
| POG | 329 | 54 | -24 | 42 | 6.41 | <0.001 |
| POG | 374 | -42 | -42 | 45 | 8.11 | <0.001 |
| ITC | 147 | -48 | -63 | -12 | 5.14 | 0.015 |
| ***RT_moral_ ∩ RT_perceptual_*** |  |  |  |  |  |  |
| MCC (extending to insula, LPFC) | 1990 | -6 | 15 | 45 | 10.98 | <0.001 |
| OC (extending to precuneus) | 458 | -36 | -84 | 30 | 7.11 | <0.001 |
| Insula (extending to LPFC) | 583 | 33 | 24 | 3 | 7.52 | <0.001 |
| Precuneus | 297 | 21 | -66 | 30 | 5.19 | 0.001 |

Note: RT_moral_, moral response time; RT_perceptual_, perceptual response time; Contrast labels indicate positive parametric associations with BOLD activity. MCC, middle cingulate cortex; LPFC, lateral prefrontal cortex; OC, occipital cortex; POG, postcentral gyrus; ITC, inferior temporal cortex; MNI, Montreal Neurological Institute; FWE, family-wise error correction.
