# Supplementary material for: Moral and perceptual conflict processing are implemented by distinct brain activation and functional connectivity patterns
Source: PLoS Biol. 2026 Oct 5;24(10):e3004025. doi: 10.1371/journal.pbio.3004025 (PMC13637876; doi:10.1371/journal.pbio.3004025)
Supplement: Supplementary file 11 [file pbio.3004025.s019.docx]

**S11 Table.** Brain regions responding parametrically to the moral or perceptual task-relevant conflict and having differential sensitivity to the moral and perceptual task-relevant conflict when task-relevant conflict, task-irrelevant conflict, and response time were all included in a general linear model (results based on GLM 1 and correct trials).

| Contrast/Region | Cluster Size | Peak MNI Coordinates | | | *T* | *P*_FWE_ |
| --- | --- | --- | --- | --- | --- | --- |
|  |  | x | y | z |  |  |
| ***RC_moral_*** |  |  |  |  |  |  |
| rpTPJ | 146 | 42 | -60 | 39 | 4.43 | 0.014 |
| DMPFC (extending to DLPFC) | 428 | 15 | 24 | 57 | 5.00 | <0.001 |
| VLPFC | 148 | 51 | 24 | 6 | 4.59 | 0.013 |
| ***RC_perceptual_*** |  |  |  |  |  |  |
| rOC (extending to SPC) | 273 | 24 | -72 | 48 | 4.79 | 0.001 |
| lOC (extending to ITC and SPC) | 456 | -39 | -84 | 9 | 6.13 | <0.001 |
| ***-RC_perceptual_*** |  |  |  |  |  |  |
| raTPJ | 128 | 60 | -48 | 33 | 4.44 | 0.023 |
| lTPJ | 148 | -54 | -42 | 36 | 5.90 | 0.013 |
| ***RC_moral_ > RC_perceptual_*** |  |  |  |  |  |  |
| rTPJ | 303 | 51 | -54 | 36 | 6.33 | <0.001 |
| lTPJ | 236 | -54 | -48 | 36 | 5.08 | 0.002 |
| DLPFC | 136 | 42 | 18 | 39 | 4.72 | 0.018 |
| VLPFC | 120 | 33 | 54 | 6 | 4.94 | 0.028 |
| ***RC_perceptual_ > RC_moral_*** |  |  |  |  |  |  |
| rOC (extending to SPC) | 180 | 36 | -75 | 6 | 4.40 | 0.006 |
| lOC (extending to ITC) | 190 | -39 | -81 | 6 | 5.03 | 0.005 |

Note: RC_moral_, moral task-relevant conflict; RC_perceptual_, perceptual task-relevant conflict; Contrast labels without a minus sign indicate positive parametric associations with BOLD activity; labels with a minus sign indicate negative parametric associations. TPJ, temporoparietal junction; DMPFC, dorsomedial prefrontal cortex; DLPFC, dorsolateral prefrontal cortex; VLPFC, ventrolateral prefrontal cortex; OC, occipital cortex; SPC, superior parietal cortex; ITC, inferior temporal cortex; r, right; l, left; a, anterior; p, posterior; MNI, Montreal Neurological Institute; FWE, family-wise error correction.
