# Supplementary material for: Moral and perceptual conflict processing are implemented by distinct brain activation and functional connectivity patterns
Source: PLoS Biol. 2026 Oct 5;24(10):e3004025. doi: 10.1371/journal.pbio.3004025 (PMC13637876; doi:10.1371/journal.pbio.3004025)
Supplement: Supplementary file 12 [file pbio.3004025.s020.docx]

**S12 Table.** Brain regions responding parametrically to the moral and/or perceptual response time and having differential sensitivity to the moral and perceptual response time when task-relevant conflict, task-irrelevant conflict, and response time were all included in a general linear model (results based on GLM 1 and correct trials).

| Contrast/Region | Cluster Size | Peak MNI Coordinates | | | *T* | *P*_FWE_ |
| --- | --- | --- | --- | --- | --- | --- |
|  |  | x | y | z |  |  |
| ***RT_moral_*** |  |  |  |  |  |  |
| MCC | 808 | -3 | 12 | 48 | 8.84 | <0.001 |
| OC | 228 | -42 | -75 | 27 | 8.57 | 0.002 |
| Precuneus | 395 | 21 | -66 | 24 | 4.89 | <0.001 |
| Insula (extending to LPFC) | 406 | 36 | 27 | 0 | 7.68 | <0.001 |
| Insula (extending to LPFC) | 768 | -30 | 27 | 3 | 8.09 | <0.001 |
| Caudate | 117 | 12 | 6 | 3 | 4.99 | 0.033 |
| ***RT_perceptual_*** |  |  |  |  |  |  |
| MCC | 287 | -6 | 9 | 51 | 8.94 | 0.001 |
| Insula (extending to LPFC) | 226 | 33 | 24 | 3 | 6.80 | 0.002 |
| Insula (extending to LPFC, POG, and precuneus) | 2159 | -30 | 24 | 3 | 8.02 | <0.001 |
| ITC | 263 | -54 | -57 | 0 | 5.31 | 0.001 |
| Thalamus | 133 | -6 | -24 | -4 | 4.38 | 0.020 |
| ***RT_moral_ > RT_perceptual_*** |  |  |  |  |  |  |
| Caudate | 149 | -15 | 24 | 24 | 4.32 | 0.012 |
| ***RT_perceptual_ > RT_moral_*** |  |  |  |  |  |  |
| POG | 334 | -51 | -33 | 48 | 5.36 | <0.001 |
| ***RT_moral_ ∩ RT_perceptual_*** |  |  |  |  |  |  |
| MCC | 269 | -6 | 12 | 48 | 7.98 | 0.001 |
| Insula (extending to LPFC) | 184 | 33 | 24 | 3 | 6.60 | 0.007 |
| Insula (extending to LPFC) | 143 | -30 | 24 | 3 | 7.43 | 0.018 |
| LPFC | 351 | -42 | 27 | 21 | 6.16 | <0.001 |

Note: RT_moral_, moral response time; RT_perceptual_, perceptual response time; Contrast labels indicate positive parametric associations with BOLD activity. MCC, middle cingulate cortex; OC, occipital cortex; LPFC, lateral prefrontal cortex; POG, postcentral gyrus; ITC, inferior temporal cortex; MNI, Montreal Neurological Institute; FWE, family-wise error correction.
