# Supplementary material for: Moral and perceptual conflict processing are implemented by distinct brain activation and functional connectivity patterns
Source: PLoS Biol. 2026 Oct 5;24(10):e3004025. doi: 10.1371/journal.pbio.3004025 (PMC13637876; doi:10.1371/journal.pbio.3004025)
Supplement: Supplementary file 13 [file pbio.3004025.s021.docx]

**S13 Table.** Brain regions showing parametric associations with moral and/or perceptual chosen value and their domain-specific sensitivity.

| Contrast/Region | Cluster Size | Peak MNI Coordinates | | | *T* | *P*_FWE_ |
| --- | --- | --- | --- | --- | --- | --- |
|  |  | x | y | z |  |  |
| ***CV_moral_*** |  |  |  |  |  |  |
| lOC (extending to calcarine cortex) | 613 | -42 | -78 | 24 | 8.21 | <0.001 |
| rOC (extending to calcarine cortex) | 437 | 42 | -69 | 12 | 6.55 | <0.001 |
| Precuneus | 104 | 12 | -45 | 54 | 4.31 | 0.040 |
| ***-CV_moral_*** |  |  |  |  |  |  |
| DMPFC | 358 | -6 | 42 | 45 | 5.51 | 0.002 |
| VLPFC | 676 | -48 | 21 | -3 | 6.60 | 0.002 |
| ***CV_perceptual_*** |  |  |  |  |  |  |
| lOC and rOC (extending to calcarine cortex) | 3043 | -12 | -99 | 0 | 13.65 | <0.001 |
| Calcarine cortex | 149 | -18 | -57 | 12 | 5.39 | 0.012 |
| lTPJ | 167 | -39 | -57 | 42 | 4.84 | 0.007 |
| ***-CV_perceptual_*** |  |  |  |  |  |  |
| MTC | 130 | 42 | -54 | 6 | 5.67 | 0.020 |
| ***CV_moral_ > CV_perceptual_*** |  |  |  |  |  |  |
| Occipitotemporal cortex | 201 | 51 | -69 | 6 | 6.61 | 0.003 |
| Occipitotemporal cortex | 179 | -54 | -69 | 3 | 5.85 | 0.005 |
| ***CV_perceptual_ > CV_moral_*** |  |  |  |  |  |  |
| lOC and rOC | 2014 | -12 | -99 | 0 | 10.39 | <0.001 |
| lTPJ | 193 | -39 | -57 | 42 | 5.54 | 0.004 |
| DLPFC | 107 | -45 | 18 | 42 | 4.93 | 0.037 |
| VLPFC | 456 | -42 | 39 | -9 | 5.40 | <0.001 |
| ***CV_moral_ ∩ CV_perceptual_*** |  |  |  |  |  |  |
| rOC | 195 | 33 | -75 | 27 | 5.19 | 0.005 |
| Calcarine cortex | 109 | -18 | -57 | 12 | 5.65 | 0.041 |

Note: CV_moral_, moral chosen value; CV_perceptual_, perceptual chosen value; Contrast labels without a minus sign indicate positive parametric associations with BOLD activity; labels with a minus sign indicate negative parametric associations. OC, occipital cortex; DMPFC, dorsomedial prefrontal cortex; VLPFC, ventrolateral prefrontal cortex; TPJ, temporoparietal junction; MTC, middle temporal cortex; DLPFC, dorsolateral prefrontal cortex; r, right; l, left; MNI, Montreal Neurological Institute; FWE, family-wise error correction.
