# Supplementary material for: Moral and perceptual conflict processing are implemented by distinct brain activation and functional connectivity patterns
Source: PLoS Biol. 2026 Oct 5;24(10):e3004025. doi: 10.1371/journal.pbio.3004025 (PMC13637876; doi:10.1371/journal.pbio.3004025)
Supplement: Supplementary file 14 [file pbio.3004025.s022.docx]

**S14 Table.** Functional connectivity responding parametrically to the moral task-relevant conflict and having differential sensitivity to the moral and perceptual task-relevant conflict.

| Contrast/Region | Seed region | Cluster Size | Peak MNI Coordinates | | | *T* | *P*_FWE_ |
| --- | --- | --- | --- | --- | --- | --- | --- |
|  |  |  | x | y | z |  |  |
| ***RC_moral_*** |  |  |  |  |  |  |  |
| OC | rpTPJ | 135 | 27 | -81 | 3 | 4.63 | 0.020 |
| OC | rpTPJ | 134 | -33 | -87 | -6 | 4.11 | 0.020 |
| OC | DMPFC | 288 | -30 | -63 | -21 | 4.63 | <0.001 |
| Precuneus | DMPFC | 373 | -21 | -57 | 54 | 5.01 | <0.001 |
| ***RC_moral_ > RC_perceptual_*** |  |  |  |  |  |  |  |
| OC | DMPFC | 235 | -36 | -81 | -12 | 5.49 | 0.001 |

Note: RC_moral_, moral task-relevant conflict; RC_perceptual_, perceptual task-relevant conflict; Contrast labels indicate positive conflict-dependent functional connectivity effects. OC, occipital cortex; rpTPJ, right posterior temporoparietal junction; DMPFC, dorsomedial prefrontal cortex; MNI, Montreal Neurological Institute; FWE, family-wise error correction.
