# Supplementary material for: Moral and perceptual conflict processing are implemented by distinct brain activation and functional connectivity patterns
Source: PLoS Biol. 2026 Oct 5;24(10):e3004025. doi: 10.1371/journal.pbio.3004025 (PMC13637876; doi:10.1371/journal.pbio.3004025)
Supplement: Supplementary file 15 [file pbio.3004025.s023.docx]

**S15 Table.** Brain activation associated with perceptual threshold.

| Contrast/Region | Parametric modulator | Cluster Size | Peak MNI Coordinates | | | *T* | *P*_FWE_ |
| --- | --- | --- | --- | --- | --- | --- | --- |
|  |  |  | x | y | z |  |  |
| ***Perceptual α*** |  |  |  |  |  |  |  |
| *ITC* | *RC_perceptual_* | 106 | -42 | -51 | -3 | 4.69 | 0.040 |
| ***Perceptual α > Moral α*** |  |  |  |  |  |  |  |
| *ITC* | *RC_perceptual_* | 110 | -42 | -57 | 0 | 4.95 | 0.036 |

Note: *α*, threshold; RC_perceptual_, perceptual task-relevant conflict; Contrast labels indicate positive associations between DDM parameters and perceptual-conflict-related regional activation. ITC, inferior temporal cortex; MNI, Montreal Neurological Institute; FWE, family-wise error correction.
