# Supplementary material for: Moral and perceptual conflict processing are implemented by distinct brain activation and functional connectivity patterns
Source: PLoS Biol. 2026 Oct 5;24(10):e3004025. doi: 10.1371/journal.pbio.3004025 (PMC13637876; doi:10.1371/journal.pbio.3004025)
Supplement: Supplementary file 16 [file pbio.3004025.s024.docx]

**S16 Table.** Functional connectivity associated with different drift diffusion model parameters.

| Contrast/Region | Seed region | Parametric modulator | Cluster Size | Peak MNI Coordinates | | | *T* | *P*_FWE_ |
| --- | --- | --- | --- | --- | --- | --- | --- | --- |
|  |  |  |  | x | y | z |  |  |
| ***Moral k*** |  |  |  |  |  |  |  |  |
| MCC (extending to precentral gyrus) | rpTPJ | *RC_moral_* | 392 | 27 | -18 | 57 | 4.43 | <0.001 |
| ***Moral α*** |  |  |  |  |  |  |  |  |
| *SPC* | rpTPJ | *RC_moral_* | 205 | 27 | -63 | 63 | 5.50 | 0.003 |
| ***Perceptual α*** |  |  |  |  |  |  |  |  |
| Cuneus | lTPJ | *RC_perceptual_* | 103 | 15 | -78 | 39 | 5.87 | 0.041 |
| ***Moral k > Perceptual k*** |  |  |  |  |  |  |  |  |
| MCC (extending to precentral gyrus) | rpTPJ | *RC_moral_* | 135 | 24 | -21 | 54 | 4.02 | 0.018 |
| ***Moral α > Perceptual α*** |  |  |  |  |  |  |  |  |
| *SPC* | rpTPJ | *RC_moral_* | 146 | 27 | -63 | 63 | 4.89 | 0.014 |

Note: *k*, drift rate scale; *α*, threshold; RC_moral_, moral task-relevant conflict; RC_perceptual_, perceptual task-relevant conflict; Contrast labels indicate positive associations between DDM parameters and conflict-dependent functional connectivity. MCC, middle cingulate cortex; rpTPJ, right posterior temporoparietal junction; SPC, superior parietal cortex; lTPJ, left temporoparietal junction; MNI, Montreal Neurological Institute; FWE, family-wise error correction.
