# Supplementary material for: Moral and perceptual conflict processing are implemented by distinct brain activation and functional connectivity patterns
Source: PLoS Biol. 2026 Oct 5;24(10):e3004025. doi: 10.1371/journal.pbio.3004025 (PMC13637876; doi:10.1371/journal.pbio.3004025)
Supplement: Supplementary file 17 [file pbio.3004025.s025.docx]

**S17 Table.** Descriptive results of the picture ratings.

| Ratings | Mean | Standard deviation | Maximum | Minimum |
| --- | --- | --- | --- | --- |
| Moral rating | 47% | 27% | 93% | 0% |
| Perceptual rating | 46% | 16% | 88% | 17% |
| Difficulty rating | 18% | 10% | 47% | 1% |
| Arousal rating | 44% | 20% | 84% | 7% |

Note: Moral rating corresponds to the question “How severe is the moral violation depicted by the picture?”; Perceptual rating corresponds to the question “How much in percent does the picture cover the gray background within the white frame?”; Difficulty rating corresponds to the question “How difficult is it to understand what is happening in the picture?”; Arousal rating corresponds to the question “How aroused do you feel while viewing the picture?” The participants’ moral ratings were unrelated to the difficulty ratings (fixed effect: *β* = 0.06 ± 0.034, *T*(53) = 1.81, *P* = 0.076, hierarchical linear mixed-effect regression).
